# Three New Multiflorane-Type Triterpenes from Pumpkin (*Cucurbita maxima*) Seeds

**DOI:** 10.3390/molecules18055568

**Published:** 2013-05-14

**Authors:** Takashi Kikuchi, Mika Takebayashi, Mayumi Shinto, Takeshi Yamada, Reiko Tanaka

**Affiliations:** Osaka University of Pharmaceutical Sciences, 4-20-1 Nasahara, Takatsuki-shi, Osaka 569-1094, Japan

**Keywords:** *Cucurbita maxima*, multiflorane-type triterpenes, melanogenesis inhibitory activity, cytotoxic activity

## Abstract

Three new multiflorane-type triterpenes; 7α-methoxymultiflor-8-ene-3α,29-diol 3-acetate-29-benzoate (**1**), 7-oxomultiflor-8-ene-3α,29-diol 3-acetate-29-benzoate (**2**), and multiflora-7,9(11)-diene-3α,29-diol 3-*p*-hydroxybenzoate-29-benzoate (**3**), were isolated from seeds of *Cucurbita maxima*, along with three known compounds. Compound **3** and multiflora-7,9(11)-diene-3α-29-diol 3-benzoate (**5**) exhibited potent inhibitory effects on melanogenesis, with low cytotoxicities, and **2** exhibited single-digit micromolar cytotoxicity against HL-60 and P388 cells.

## 1. Introduction

Pumpkins, including *Cucurbita moschata*, *C. pepo*, and *C. maxima* are gourd squashes of the genus *Cucurbita* and the family Cucurbitaceae.* Cucurbita moschata* seeds have been used as an anthelmintic [1], and *Cucurbita pepo* seeds as an anthelmintic and a diuretic [2].

*Cucurbita maxima* (English name: squash, pumpkin, Japanese name: kabocha) is indigenous to the plateaus of central and south America, but is cultivated throughout the World. Its fruits, flowers, and seeds have been eaten as vegetables containing vitamins A, C, and E. Several triterpenes such as cucurbita-5,24-dienol [3] and α− and β-amyrin [4] are present in the seeds of *Cucurbita maxima*. Additionally, it has been demonstrated that the seeds and flowers of *C. maxima* contain sterols [4,5,6]. Herein, we report the isolation and structural elucidation of three new multiflorane-type triterpenes along with three known compounds, multiflora-7,9(11)-diene-3α,29-diol 3,29-dibenzoate (**4**), multiflora-7,9(11)-diene-3α-29-diol 3-benzoate (**5**) and multiflora-5,7,9(11)-triene-3α,29-diol 3,29-dibenzoate (**6**), from seeds of *C. maxima* and describe their inhibitory effects on α-MSH-induced melanogenesis in B16 melanomas, and cytotoxic activities against the HL-60 and P388 leukemia cell lines.

## 2. Results and Discussion

**Figure 1:**
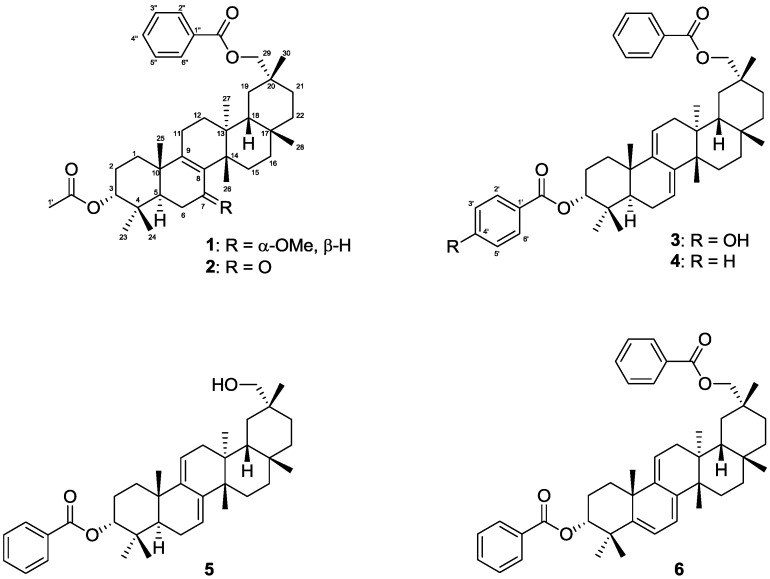
Chemical structures of isolated compounds **1**–**6**.

Three new multiflorane-type triterpenes **1**–**3** and three known multiflorane-type triterpenes **4**–**6** were isolated from the MeOH extract of *C. maxima* seeds (Figure 1).

**Figure 2:**
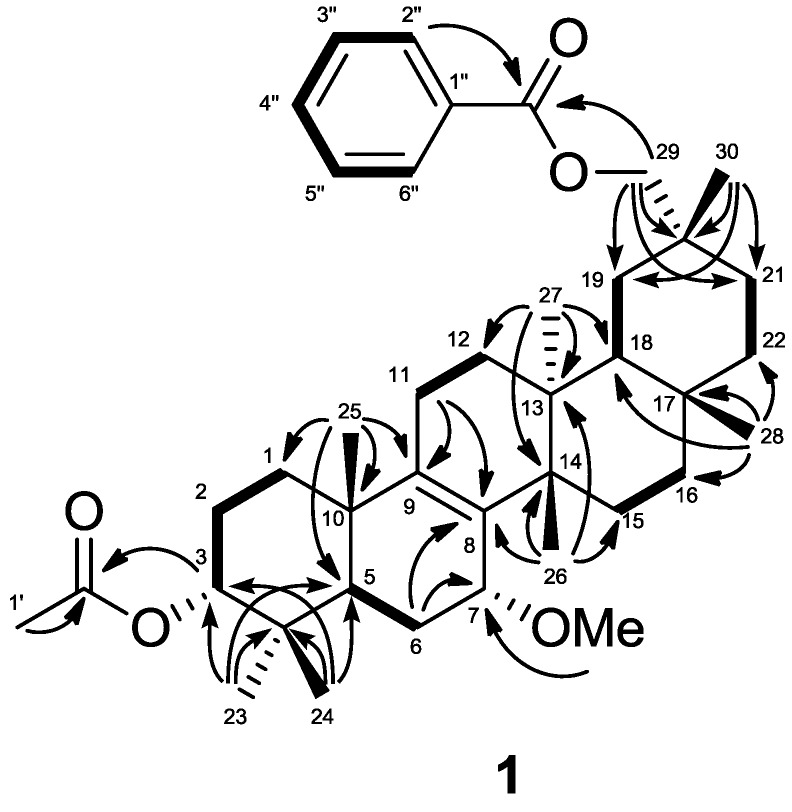
Key HMBC (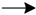) and ^1^H-^1^H COSY (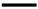) correlations of **1**.

The compound **1** was obtained as an amorphous solid with a molecular ion at *m/z* 618.4282 [M]^+^ (calcd. for C_40_H_58_O_5_, 618.4285) in the HREIMS. The IR spectrum showed absorptions indicating two carbonyl groups [ν_max_ 1743 (C=O), 1724 (C=O), 1267 (C–O), 1247 (C–O) cm^−1^]. The ^1^H- and ^13^C-NMR spectra (δ_H_ and δ_C_ in ppm, Table 1) displayed signals for seven tertiary methyl groups [δ_H_ 0.88, 0.93, 0.94, 1.04, 1.06, 1.08, 1.13 (each s)], an oxymethylene [δ_H_ 4.11, 4.15 (each d); δ_C_ 73.0 (t)], two oxymethines [δ_H_ 3.53 (brs), 4.68 (t); δ_C_ 74.0 (d), 77.2 (d)], a tetrasubstituted olefin [δ_C_ 135.2 (s), 140.0 (s)], an acetoxy group [2.06 (s); δ_C_ 21.5 (q), 171.1 (s)], a benzoyl group [δ_H_ 7.46 (2H, tt), 7.57 (1H, tt), 8.06 (2H, dd); δ_C_ 128.3 (d), 129.5 (d), 130.7 (s), 132.7 (d), 166.6 (s)], and a methoxyl group [δ_H_ 3.24 (s); 55.0 (q)]. In the HMBC experiment (Figure 2), the following correlations were observed: Me-23 [δ_H_ 0.88 (s)] to C-3 [δ_c_ 77.2 (d)], C-4, C-5, and C-24; Me-24 [δ_H_ 0.93 (s)] to C-3, C-4, C-5, and Me-23; Me-25 [δ_H_ 0.94 (s)] to C-1, C-5, C-9 [δ_C_ 140.0 (s)], and C-10; Me-26 [δ_H_ 1.04 (s)] to C-8 [δ_C_ 135.2 (s)], C-13, C-14, and C-15; Me-27 [δ_H_ 1.06 (s)] to C-12, C-13, C-14, and C-18; Me-28 [δ_H_ 1.13 (s)] to C-16, C-17, C-18, and C-22; H_2_-29 [δ_H_ 4.11, 4.15 (each d)] to C-19, C-20, C-21, C-30, and 29-OCO [δ_C_ 166.6 (s)]; Me-30 [δ_H_ 1.08 (s)] to C-19, C-20, C-21, and C-29 [δ_C_ 73.0 (t)]; H-3 [δ_H_ 4.68 (t)] to 3-OCO [δ_C_ 171.1 (s)]; H-5, H-6β, and 7-OMe [δ_H_ 3.24 (s)] to C-7 [δ_C_ 74.0 (d)]; H-6β, H-11, and Me-26 to C-8 [δ_C_ 135.2 (s)]; and H-11 and Me-25 to C-9 [δ_C_ 140.0 (s)] (Figure 2).

**Table 1:** ^1^H (500 MHz) and ^13^C (125 MHz) NMR spectroscopic data of compounds **1**–**3** (CDCl_3_) ^a^.

Position	1		2		3	
	δ_C_ (ppm), type	δ_H_ (ppm) (*J* in Hz)	δ_C_ (ppm), type	δ_H_ (ppm) (*J* in Hz)	δ_C_ (ppm), type	δ_H_ (ppm) (*J* in Hz)
1	29.7, t	α, 1.39, m	29.5, t	1.59, m	31.8, t	α, 1.97, m
		β, 1.45, m				β, 1.58 m
2	23.4, t	α, 1.64, m	22.9, t	α, 1.75, m	23.1, t	α, 1.87, m
		β, 1.85, m		β, 1.95, m		β, 1.98, m
3	77.2, d	4.68, t (2.8)	76.9, d	4.71, t (2.5)	78.8, d	4.82, brd (3.2)
4	36.2, s		36.5, s		37.6, s	
5	39.7, d	1.99, dd (12.5, 1.1)	42.6, d	2.07, dd (7.5, 3.9)	43.9, d	1.94, m
6	22.4, t	α, 1.89, m	36.2, t	2.35, m	23.7, t	α, 2.14, brt (5.0)
		β, 1.30, m				β, 2.08, m
7	74.0, d	3.53, brs	198.3, s		119.4, d	5.60, brd (5.9)
8	135.2, s		142.5, s		142.3, s	
9	140.0, s		163.3, s		145.8, s	
10	38.5, s		39.2, s		36.4, s	
11	21.0, t	1.95, 2H, m	22.2, t	α, 2.30, m	114.8, d	5.29, brd (5.9)
				β, 2.14, m		
12	31.2, t	α, 1.34, m	29.8, t	α, 1.38, m	39.1, t	α, 2.08, m
		β, 1.60, m		β, 1.59, m		β, 1.79, m
13	37.0, s		38.0, s		37.5, s	
14	41.7, s		39.1, s		40.4, s	
15	25.4, t	α, 2.18, m	29.4, t	α, 2.43, m	27.6, t	α, 1.63, m
		β, 1.25, m		β, 1.59, m		β, 1.42, m
16	36.9, t	1.56, 2H, m	35.9, t	α, 1.39, m	37.2, t	α, 1.76, m
				β, 1.63, m		β, 1.49, m
17	31.1, s		31.1, s		31.9, s	
18	44.0, d	1.59, m	41.3, d	1.66, m	45.1, d	1.68, m
19	28.8, t	α, 1.84, m	30.2, t	α, 1.63, m	29.6, t	α, 1.76, m
		β, 1.30, m		β, 1.29, dd (15.7, 3.9)		β, 1.30, m
20	31.9, s		32.4, s		29.9, s	
21	29.5, t	1.51, 2H, m	28.3, t	α, 1.56, m	30.1, t	1.63, 2H, m
				β, 1.47, m		
22	35.7, t	α, 1.79, m	38.5, t	α, 1.50, m	33.0, t	α, 1.89, m
		β, 0.97, m		β, 1.03, m		β, 0.95, m
23	27.2, q	0.88, s	26.7, q	0.87, s	28.0, q	0.90, s
24	22.3, q	0.93, s	21.4, q	0.99, s	21.6, q	1.03, s
25	18.2, q	0.94, s	18.0, q	1.03, s	21.1, q	1.01, s
26	26.1, q	1.04, s	26.7, q	1.39, s	21.2, q	0.94, s
27	18.9, q	1.06, s	18.3, q	0.99, s	19.9, q	1.03, s
28	31.2, q	1.13, s	30.6, q	1.22, s	31.4, q	1.11, s
29	73.0, t	a, 4.15, d (10.8)	75.0, t	a, 4.05, d (10.5)	74.2, t	a, 4.34, d (10.7)
		b, 4.09, d (10.8)		b, 4.02, d (10.5)		b, 4.12, d (10.7)
30	29.7, q	1.08, s	26.5, q	1.15, s	31.3, q	1.16, s
3-OCO	171.1, s		170.6, s		165.4, s	
1'	21.5, q	2.06, s	21.3, q	2.07, s	123.1, s	
2', 6'					131.9, d	7.85, 2H, dd (8.4, 2.8)
3', 5'					115.3, d	6.84, 2H, dd (8.4, 2.8)
4'					160.8, s	
29-OCO	166.6, s		166.8, s		168.5, s	
1''	130.7, s		130.6, s		130.2, s	
2'', 6''	129.5, d	8.06, 2H, dd (7.4, 1.3)	129.5, d	8.06, 2H, dd (8.0, 1.4)	129.6, d	8.04, 2H, dd (7.4, 1.4)
3'', 5''	128.3, d	7.46, 2H, tt (7.4, 1.3)	132.8, d	7.46, 2H, tt (8.0, 1.4)	128.8, d	7.46, 2H, tt (7.4, 1.4)
4''	132.7, d	7.57, tt (7.4, 1.3)	128.4, d	7.58, tt (8.0, 1.4)	133.6, d	7.56, tt (7.4, 1.4)
7-OMe	55.0, q	3.24, s				
4'-OH						7.49, brs

^a^ Assignments were based on ^1^H-^1^H COSY, HMQC, HMBC and NOESY spectroscopic data.

**Figure 3:**
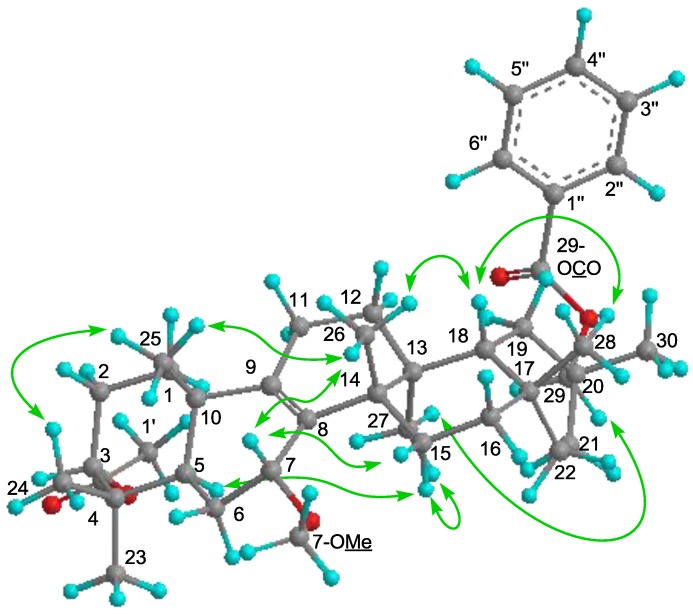
Key NOE (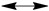) correlations of **1**.

In the ^1^H-^1^H COSY experiment, H-7 [δ_H_ 3.53 (brs)] correlated with H_2_-6 [δ_H_ 1.30, 1.89]. The following significant NOE interactions were observed in **1**: H-5/H-15α; H-15α/Me-27; Me-27/H_2_-29; Me-26/H-7, Me-25, and H-18; H-18/Me-28 (Figure 3). Therefore, the methoxy group at C-7 had the α (axial)-orientation. The configuration of the acetoxy group at C-3 was established as the α (axial)-orientation due to the NOE correlations between H-3 and Me-23 and Me-24, and the coupling constants of H-3 [δ_H_ 4.68 (t, *J*_3__β.2__α;3__β,2__β_ = 2.8 Hz)]. Therefore, **1** was determined as 7α-methoxymultiflor-8-ene-3α,29-diol 3-acetate-29-benzoate.

**Figure 4:**
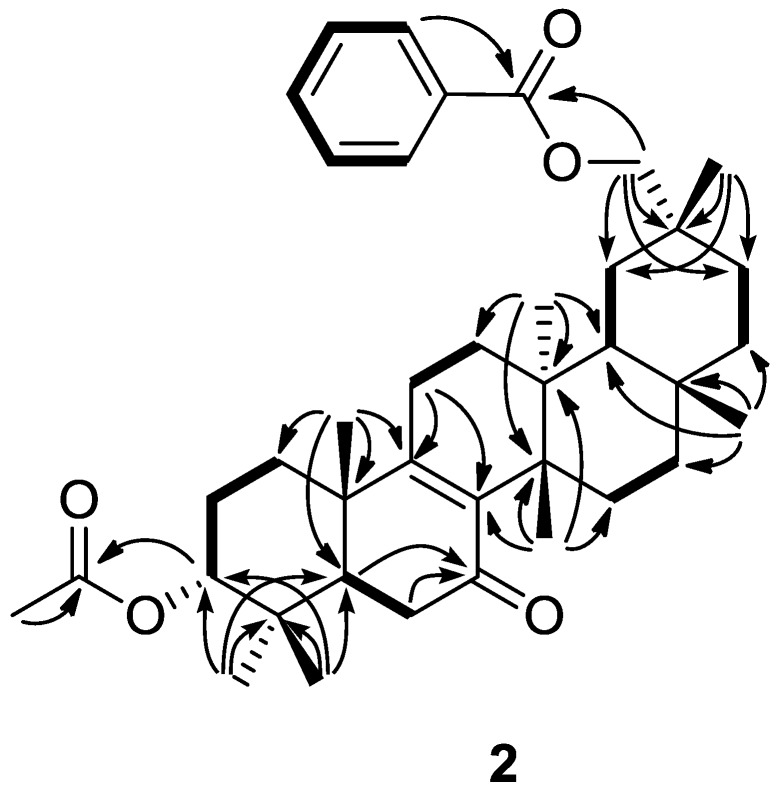
Key HMBC (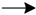) and ^1^H-^1^H COSY (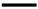) correlations of **2**.

Compound **2** exhibited a [M]^+^ ion in the HREIMS data at *m/z* 602.3975 whose molecular formula was C_39_H_54_O_5_(calcd. 602.3972). The IR and UV spectra showed absorptions indicating two carbonyl groups [ν_max_ 1739 (C=O), 1723 (C=O), 1270 (C−O), 1245 (C−O) cm^−1^] and an α,β-unsaturated six-membered ring ketone [ν_max_ 1658 cm^−1^; λ_max_ 233.0 nm (log ε 3.91)]. **2** is similar to **1** according to the ^1^H- and ^13^C-NMR spectra (δ_H_ and δ_C_ in ppm). In the HMBC experiment, cross-peaks were observed from H-5 and H-6 to C-7 [δ_C_ 198.3 (s)]; and from H_2_-11 to C-8 [δ_C_ 142.5 (s)] and C-9 [δ_C_ 163.3 (s)] (Figure 4). In the ^1^H-^1^H COSY experiment, H_2_-11 [δ_H_ 2.14, 2.30] correlated with H_2_-12 [δ_H_ 1.38, 1.59], but H_2_-6 [δ_H_ 2.35 (2H)] correlated with only H-5 [δ_H_ 2.07 (dd)] (Figure 4). NOESY experiments revealed that the relative of **2** to have the same conformation as **1**. As a result, **2** was determined to be 7-oxomultiflor-8-ene-3α,29-diol 3-acetate-29-benzoate.

**Figure 5:**
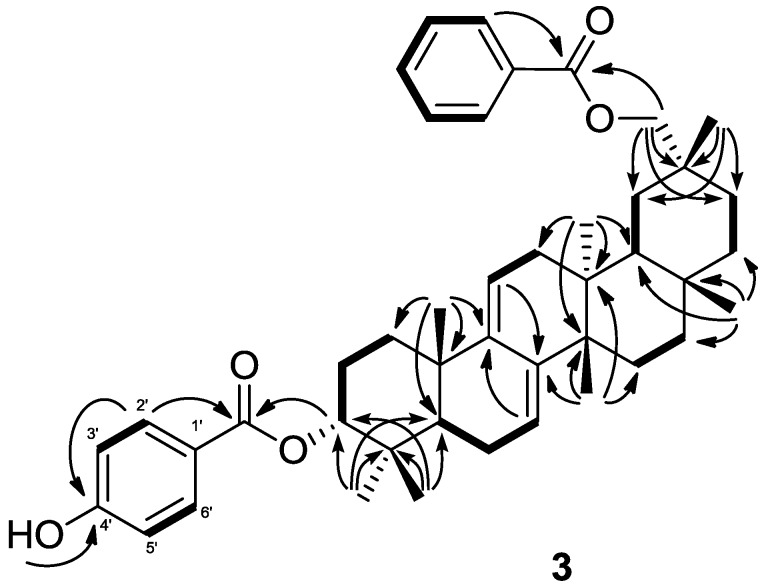
Key HMBC (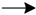) and ^1^H-^1^H COSY () correlations of **3**.

The molecular formula of **3** was determined as C_44_H_56_O_5_ based on the HREIMS (*m/z* 664.4127, calcd. 664.4127). In addition, *m/z* 526 [M−C_7_H_6_O_3_]^+^ indicated the presence of a hydroxybenzoyloxy group. The IR spectrum showed the existence of a hydroxy group (ν_max_3436 cm^−1^) and aryl esters (ν_max_ 1716, 1683, 1509, 1456, 1274 cm^−1^). The ^1^H- and ^13^C NMR spectra of **3** displayed signals for seven tertiary methyl groups [δ_H_ 0.90, 0.94, 1.01, 1.03 (6H), 1.11, 1.16 (each s)], an oxymethylene [δ_H_ 4.12, 4.34 (each d); δ_C_ 74.2 (t)], an oxymethine [δ_H_ 4.82 (brd); δ_C_ 78.8 (d)], a heteroannular diene [δ_H_ 5.29, 5.60 (each brd); δ_C_ 114.8 (d), 119.4 (d), 142.3 (s), 145.8 (s)], two aryl ester groups [δ_H_ 6.84 (dd), 7.46 (tt), 7.56 (tt), 7.85 (dd), 8.04 (dd); δ_C_ 115.3 (d), 123.1 (s), 128.8 (d), 129.6 (d), 130.2 (s), 131.9 (d), 133.6 (d), 160.8 (s), 165.4 (s), 168.5 (s)], and a hydroxyl group [δ_H_ 7.49 (brs)]. The ^1^H and ^13^C-NMR spectra of **3** were similar to those of multiflora-7,9(11)-diene-3α-29-diol 3,29-dibenzoate (**4**) except for the signal of the C-4' [δ_C_ 160.8 (s) in **3**, δ_C_ 133.6 (s) in **4**]. In the HMBC experiment, the correlations were observed from 4'-OH [δ_H_ 7.49 (brs)] to C-4' (Figure 5). Therefore the structure of **3** was determined to be multiflora-7,9(11)-diene-3α,29-diol 3-*p*-hydroxybenzoate-29-benzoate. 

The known compounds **4** [7,8] and **5** [9] were identified by comparing MS and ^1^H and ^13^C-NMR data with published data, and **6** [7] by MS and ^1^H NMR data_._

The six multiflorane triterpenes **1**−**6** from *C. maxima* were evaluated for inhibitory activities against α-MSH-induced melanogenesis in B16 melanomas (Table 2). At a low concentration (10 μM), **5** inhibited melanogenesis (76.9% of melanin content) with low cytotoxicity (99.5% of cell viability). **5** also inhibited melanogenesis (70.9% of melanin content) with low cytotoxicity (97.7% cell viability) at 30 μM. At a high concentration (100 μM), **3** and **5** exhibited inhibitory activities (51.8 and 67.4% of melanin content, respectively) with low cytotoxicity (95.1 and 99.6% of cell viability, respectively). The activity levels of compounds **5** at 10 and 30 μM were comparable with or superior to those of the positive control, arbutin, which has been recognized as a useful depigmentation compound for skin whitening in the cosmetic industry [10]. It appears that two multiflorane-type triterpenes, **5** from *C. maxima* seeds, may be valuable as potential skin-whitening agents. The melanogenesis inhibitory activity of **2** (28.1% of melanin content at 100 μM) is thought to be due to their cytotoxic action (69.0% of cell viability at 100 μM).

**Table 2:** Melanogenesis inhibitory activity and cytotoxicity in B16 mouse melanoma cells of multiflorane-type triterpenes isolated from *Cucurbita maxima* seeds ^a^.

Compound	mean ± S.D. (%) at 10 μM		mean ± S.D. (%) at 30 μM		mean ± S.D. (%) at 100 μM	
	Melanin content	Cell viability	Melanin content	Cell viability	Melanin content	Cell viability
**1**	94.8 ± 0.5	92.7 ± 2.2	77.1 ± 3.8	84.4 ± 4.0	73.7 ± 3.6	84.3 ± 5.2
**2**	106.8 ±9.3	106.3 ± 8.0	92.2 ± 5.4	107.1 ± 7.4	28.1 ± 2.3	69.0 ± 4.5
**3**	91.2 ± 2.2	107.2 ± 5.1	81.8 ± 4.0	105.6 ± 3.1	51.8 ± 8.0	95.1 ± 4.3
**4**	98.4 ± 3.2	110.8 ± 4.3	102.2 ± 11.7	103.0 ± 8.2	95.4 ± 8.4	101.1 ± 5.9
**5**	76.9 ± 4.0	99.5 ± 3.3	70.9 ± 0.1	97.7 ± 3.1	67.4 ± 3.6	99.6 ± 2.0
**6**	107.8 ± 2.6	91.0 ± 1.6	111.8 ± 7.1	81.8 ± 2.1	82.0 ± 5.1	74.4 ± 3.2
arbutin ^b^	88.9 ± 2.3	100.0 ± 2.7	72.3 ± 3.1	94.4 ± 1.2	55.3 ± 1.0	89.9 ± 0.3

^a^ Melanin content (%) and cell viability (%) were determined based on the absorbance at 450 nm, and 540 nm, respectively, by comparison with values for DMSO (100%). Each value represents the mean ± standard deviation (S.D.) of three determinations. The concentration of DMSO in the sample solution was 2 μL/mL. ^b^ Reference compound.

Six triterpenes and a reference compound, 5-fluorouracil (5-FU), were also evaluated for cytotoxic activities against human leukemia (HL-60) and murine leukemia (P388) cell lines by means of the MTT assay (Table 3). Compound **2** exhibited single-digit micromolar cytotoxicity with IC_50_ values of 7.0 and 9.5 μM against HL-60 and P388 cells, respectively. It was slightly less cytotoxic than 5-FU [IC_50_ 2.3 (HL-60); 1.9 (P388) μM] (Table 3).

**Table 3:** Cytotoxic activities of multiflorane-type triterpenes from *Cucurbita maxima* seeds.

Compound	IC_50_ (μM) ^a^	
	HL-60	P388
	(human leukemia)	(murine leukemia)
**1**	>100	>100
**2**	7.0 ± 1.1	9.5 ± 1.1
**3**	55.9 ± 1.1	92.6 ± 1.3
**4**	>100	>100
**5**	>100	>100
**6**	54.1 ± 1.3	46.7 ± 1.2
5-fluorouracil ^b^	2.3 ± 0.2	1.9 ± 0.2

^a^ HL-60 and P388 cell lines (each 1 × 10^4^ cells in 100 μL) were treated with test compounds for 72 h, and MTT solution was added to the wells. The grown cells were labeled with 5 mg/mL MTT in phosphate-buffered saline (PBS), and the absorbance of formazan dissolved with 20% sodium dodecyl sulfate (SDS) in 0.1 N HCl was measured at 550 nm using a microplate reader. Data are expressed as mean ± S.D. (n = 3); ^b^ Reference compound.

## 3. Experimental

### 3.1. General Procedures

Chemicals and reagents were purchased as follows: fetal bovine serum (FBS) from Invitrogen Co. (Carlsbad, CA, USA), 3-(4,5-dimethyl-2-thiazolyl)-2,5-diphenyl-2H-tetrazolium bromide (MTT) from Sigma-Aldrich Japan Co. (Tokyo, Japan), and 5-fluorouracil (5-FU) (purity ≥ 98.5%), arbutin (purity ≥ 95.0%), Roswell Park Memorial Institute (RPMI) 1640 medium, Dulbecco’s modified Eagle’s medium (D-MEM), and antibiotics from Nacalai Tesque, Inc. (Kyoto, Japan). All other chemicals and reagents were of analytical grade. Melting points were determined on a Yanagimoto micro-melting point apparatus and are uncorrected. Optical rotations were measured with a JASCO DIP-1000 digital polarimeter. IR spectra were recorded on a Perkin-Elmer 1720X FTIR spectrophotometer. The ^1^H (500 MHz) and ^13^C (125 MHz) NMR spectra were recorded on a Varian INOVA 500 spectrometer in CDCl_3_ with tetramethylsilane as the internal standard. The EIMS was recorded on a Hitachi 4000H double-focusing mass spectrometer (70 eV). Silica gel (70–230 mesh, Merck) and silica gel 60 (230–400 mesh, Nacalai Tesque, Inc., Kyoto, Japan) were used for column chromatography and medium-pressure liquid chromatography, respectively. The 20% AgNO_3_/SiO_2_ (w/w) used for chromatography was prepared from silica gel 60 and AgNO_3_ (Nacalai Tesque, Inc., Kyoto, Japan). HPLC was carried out on an SiO_2_ column (*Cosmosil 5SL-II column*, 25 cm × 20 mm i.d., Nacalai Tesque, Inc., Kyoto, Japan) at 25 °C with *n*-hexane/EtOAc [10:1 (HPLC system I) and 5:1 (HPLC system II), flow rate 8.0 mL/min].

### 3.2. Plant Material

The seeds of *Cucurbita maxima*, produced in Japan (Nara prefecture), were purchased from JA (Japan Agricultural Co-operation)-Takatsuki in 2011. A voucher specimen was deposited in the Herbarium of the Laboratory of Medicinal Chemistry, Osaka University of Pharmaceutical Sciences.

### 3.3. Extraction and Isolation

The seeds of *Cucurbita maxima* (3 kg) were subjected to extraction with MeOH (10 L) under reflux (1 week, 4 times). After concentration the MeOH extract (102.2 g) was then partitioned between Et_2_O and H_2_O. The Et_2_O-soluble fraction (62.2 g) was subjected to SiO_2_ column chromatography (CC) [SiO_2_ (1.5 kg); CHCl_3_/EtOAc 1:0, 5:1, 2:1, 0:1 and MeOH, in increasing order of polarity] resulting in seven fractions (Fr. A–G). Fr. B, eluted with CHCl_3_, was subjected to SiO_2_ CC to yield 10 fractions, B1–B10. Among them, Fr. B3, eluted with hexane/EtOAc (5:1), was subjected to SiO_2_ CC to yield 11 fractions; B3-1–B3-11. Preparative HPLC of B3-4 (123.0 mg), eluted with hexane/EtOAc (5:1), gave **4** (15.5 mg; t_R_ 11.2 min) (HPLC system *I*). Fr. C, eluted with CHCl_3_, was subjected to SiO_2_ CC to yield 22 fractions, C1–C22. Preparative HPLC of C3 (14.8 mg), eluted with hexane/EtOAc (10:1), gave **4** (5.1 mg) and **6** (3.1 mg; t_R_ 12.0 min), respectively (HPLC system *I*). Fr. C11 (1.3 g), eluted with hexane/EtOAc (10:1), was subjected to CC with 20% AgNO_3_/SiO_2_ to give C11-1–C11-11, followed by CC of C11-4 (795.6 mg), eluted with hexane/CHCl_3_ (20:1), with 20% AgNO_3_/SiO_2_ to yield C11-4-1–C11-4-9. Preparative HPLC of C11-4-3 (15.0 mg), eluted with hexane/EtOAc (2:1), gave **2** (2.0 mg; t_R_ 18.9 min) (HPLC system *II*). Fr. D, eluted with CHCl_3_, was fractionated with SiO_2_ CC to D1–D16. Fr. D4 (1369.0 mg), eluted with hexane/EtOAc (5:1) was subjected to SiO_2_ CC to yield D4-1–D4-12. Preparative HPLC of D4-5 (10.0 mg), eluted with hexane/EtOAc (5:1), gave **1** (1.6 mg; t_R_ 15.3 min) (HPLC system *II*). Fr. D4-7 (194.3 mg), eluted with hexane/EtOAc (5:1), was subjected to SiO_2_ CC to yield D4-7-1–D4-7-7, followed by preparative HPLC of D4-7-2 (99.9 mg), eluted with hexane/EtOAc (5:1), for the isolation of **2** (3.0 mg; t_R_ 22.2 min) and **1** (1.4 mg) (HPLC system *II*). Fr. D6 (265.4 mg), eluted with hexane/EtOAc (5:1) was subjected to SiO_2_ CC to yield D6-1–D6-11, followed by preparative HPLC of D6-2 (6.7 mg), eluted with hexane/EtOAc (10:1), for the isolation of **5** (3.6 mg; t_R_ 35.6 min) (HPLC system *I*). Fr. D7 (176.3 mg), eluted with hexane/EtOAc (10:1), was subjected to SiO_2_ CC with hexane/EtOAc (10:1) for fractionation to D7-1–D7-12. SiO_2_ CC of D7-8 gave **3** (1.7 mg).

*7α-Methoxymultiflor-8-ene-3α,29-diol 3-acetate-29-benzoate* (**1**). Amorphous solid; [α]22 *D* −74.8 (*c* 0.1, CHCl_3_); UV (EtOH) λ_max_ (logε) 206.0 (3.58), 228.5 (3.68), 271 (2.81) nm; IR (KBr) ν_max_: 2,924, 2,855, 1,743, 1,724, 1,467, 1,452, 1,374, 1,267, 1,247, 1,110, 1,072 cm^−1^; ^1^H- and ^13^C-NMR spectroscopic data (in ppm), see Table 1; EIMS *m/z* 618 [M]^+^ (10), 586 (26), 526 (30), 511 (100), 483 (9), 389 (13), 105 (66); HREIMS *m/z* 618.4282 (calcd for C_40_H_58_O_5_, 618.4285).

*7-Oxomultiflor-8-ene-3α,29-diol 3-acetate-29-benzoate* (**2**). Amorphous solid; [α]22 *D* −67.4 (*c* 0.1, CHCl_3_); UV (EtOH) λ_max_ (logε) 233.0 (3.91), 250.5 (3.81) nm; IR (KBr) ν_max_: 2,937, 2,883, 1,739, 1,723, 1,658, 1,270, 1,245 cm^−1^; ^1^H- and ^13^C-NMR spectroscopic data (in ppm), see Table 1; EIMS *m/z* 602 [M]^+^ (100), 587 (11), 542 (8), 480 (14), 465 (16), 420 (7), 371 (4), 325 (10), 303 (11), 278 (47), 243 (33), 203 (34), 105 (39); HREIMS *m/z* 602.3975 (calcd for C_39_H_54_O_5_, 602.3972).

*Multiflora-7,9(11)-diene-3α,29-diol 3-p-hydroxybenzoate-29-benzoate* (**3**). Amorphous solid; [α]22 *D* −3.9 (*c* 0.09, CHCl_3_); UV (EtOH) λ_max_ (logε) 231.0 (4.25), 246.5 (4.12), 266.5 (3.95) nm; IR (KBr) ν_max_: 3,436, 2,941, 2,863, 1,716, 1,683, 1,636, 1,509, 1,456, 1,384, 1,274, 1,166, 1,111 cm^−1^; ^1^H- and ^13^C-NMR spectroscopic data (in ppm), see Table 1; EIMS *m/z* 664 (16) [M]+, 526 (90) [M−C_7_H_6_O_3_]+, 511 (32), 389 (26), 253 (41), 121 (100); HREIMS *m/z* 664.4127 (calcd for C_44_H_56_O_5_, 664.4127).

### 3.4. Cell Cultures

The cell lines HL-60 (human leukemia) and P388 (murine leukemia) were grown in RPMI 1640 medium, while B16 4A5 cells were grown in D-MEM. The medium was supplemented with 10% FBS and antibiotics (100 units/mL penicillin and 100 μg/mL streptomycin). The cells were incubated at 37 °C in a 5% CO_2_ humidified incubator.

### 3.5. Determination of B16 4A5 Cells Proliferation

B16 4A5 cell proliferation was examined according to a method reported previously [11] with slight modifications. Briefly, B16 4A5 cells (obtained from the Riken Cell Bank, Tsukuba, Ibaraki, Japan) (3 × 10^4^ cells in 500 μL), preincubated for 24 h were treated for 48h with test samples dissolved in dimethyl sulfoxide (DMSO) at a final concentration of 100, 30, or 10 μM, and MTT solution was added. After 3 h of incubation, 2-propanol containing 0.08 M HCl was added to dissolve the formazan produced in the cells. The absorbance of each well was read at 550 nm using a microplate reader.

### 3.6. Assay of Melanin Content

The assay of melanin content was performed as described previously [11] with small modifications. B16 4A5 cells (3 × 10^4^ cells in 500 μL) were pre-incubated as above in α-MSH (100 nM)-containing medium. Test samples dissolved in DMSO were added to the medium and the cells were cultured for 48 h. The medium was removed and the cells were dissolved in 2 M NaOH containing 10% DMSO. The amount of melanin was determined spectrophotometrically by measuring absorbance at 450 nm using a microplate reader. The optical density of control cells was assumed to be 100%.

### 3.7. Cytotoxicity Assay against Cancer Cell Lines

The cytotoxicity assay against HL-60 and P388 cells was determined as described previously [12].

## 4. Conclusions

Six multiflorane-type triterpenes, including the three new compounds 7α-methoxymultiflor-8-ene-3α,29-diol 3-acetate-29-benzoate (**1**), 7-oxomultiflor-8-ene-3α,29-diol 3-acetate-29-benzoate (**2**) and multiflora-7,9(11)-diene-3α,29-diol 3-*p*-hydroxybenzoate-29-benzoate (**3**) were isolated from the MeOH extract of *Cucurbita maxima* seeds. The seeds included more **4** than other multiflorane-type triterpenes. It was suggested that multiflorane-type triterpenes in *C. maxima* were biosynthesized from **4**, or consumed to biosynthesize **4**. The melanogenesis inhibitory activity of **5** suggests they may be potential skin whitening agents. On the other hand, the results of cytotoxicity assays suggest that **2** may be valuable as an anticancer lead compound.

## Supplementary Materials

Supplementary materials can be accessed at: http://www.mdpi.com/1420-3049/18/5/5568/s1.
