# Supplementary material for: Three New Multiflorane-Type Triterpenes from Pumpkin (Cucurbita maxima) Seeds
Source: Molecules. 2013 May 14;18(5):5568–79. doi: 10.3390/molecules18055568 (PMC6270121; doi:10.3390/molecules18055568)

Supplementary Materials

Figure S1. ^{1}H-NMR spectrum of compound **1**.

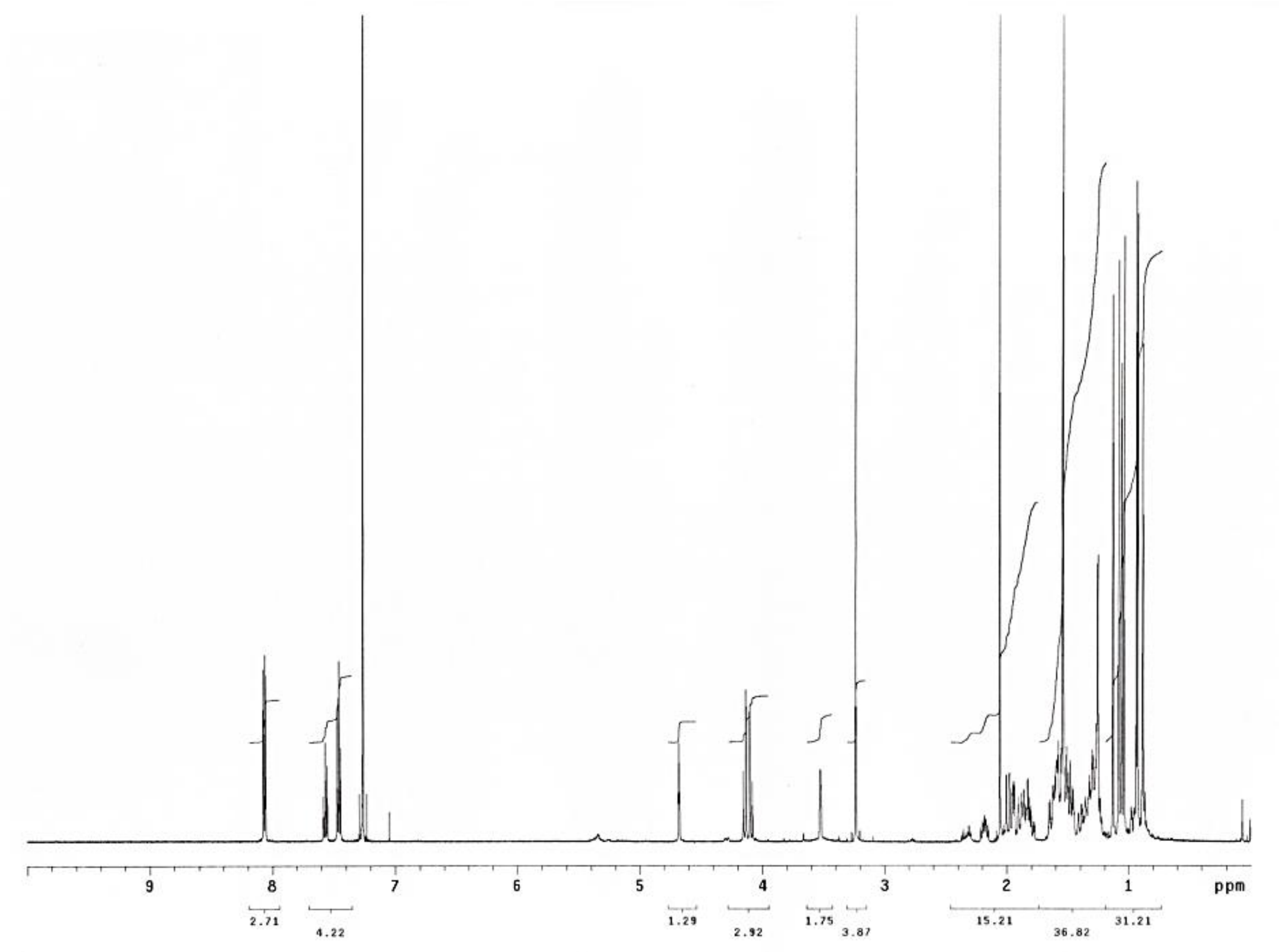

Figure S2. ^{13}C-NMR spectrum of compound **1**.

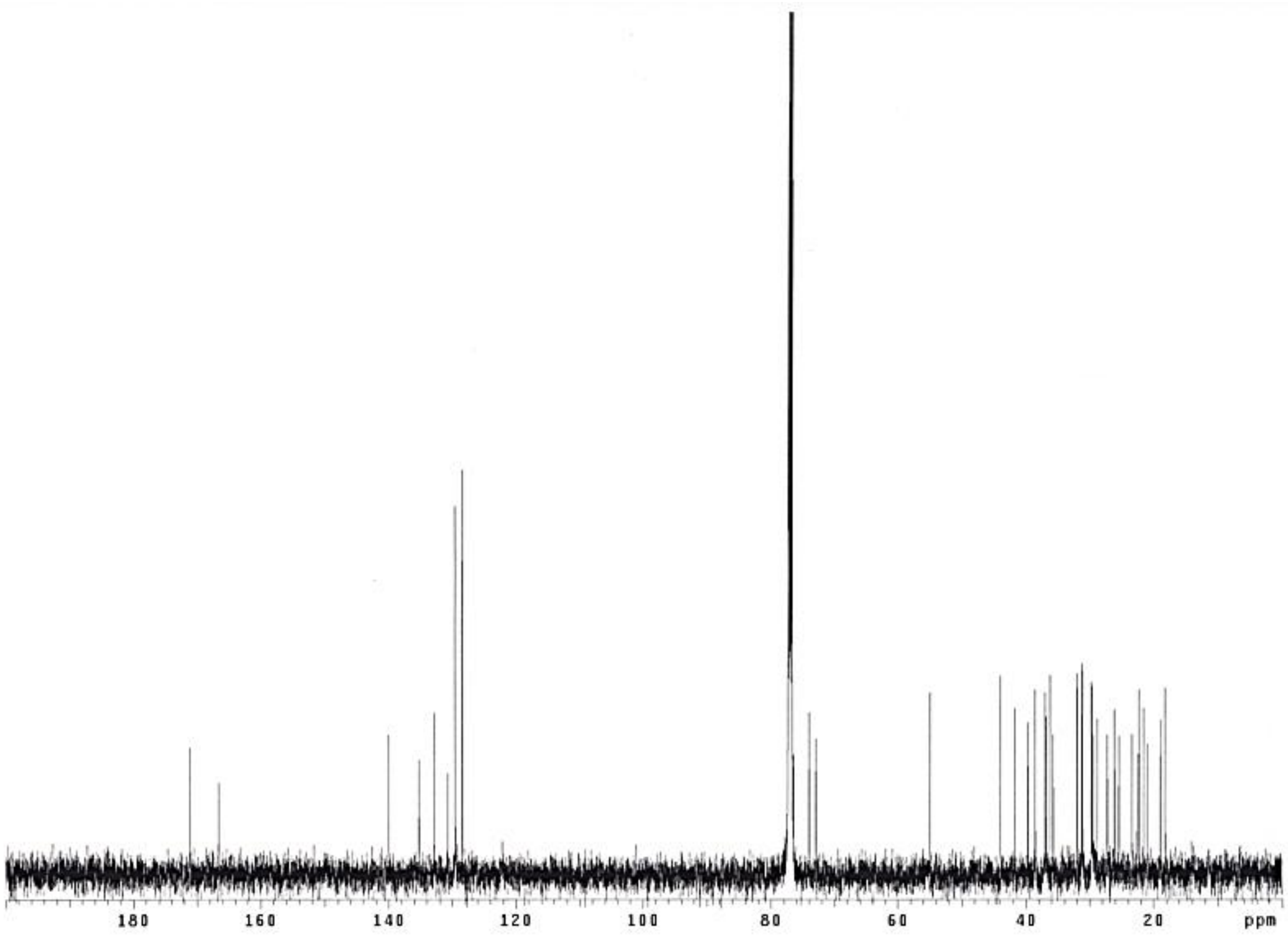

Figure S3. HSQC spectrum of compound **1**.

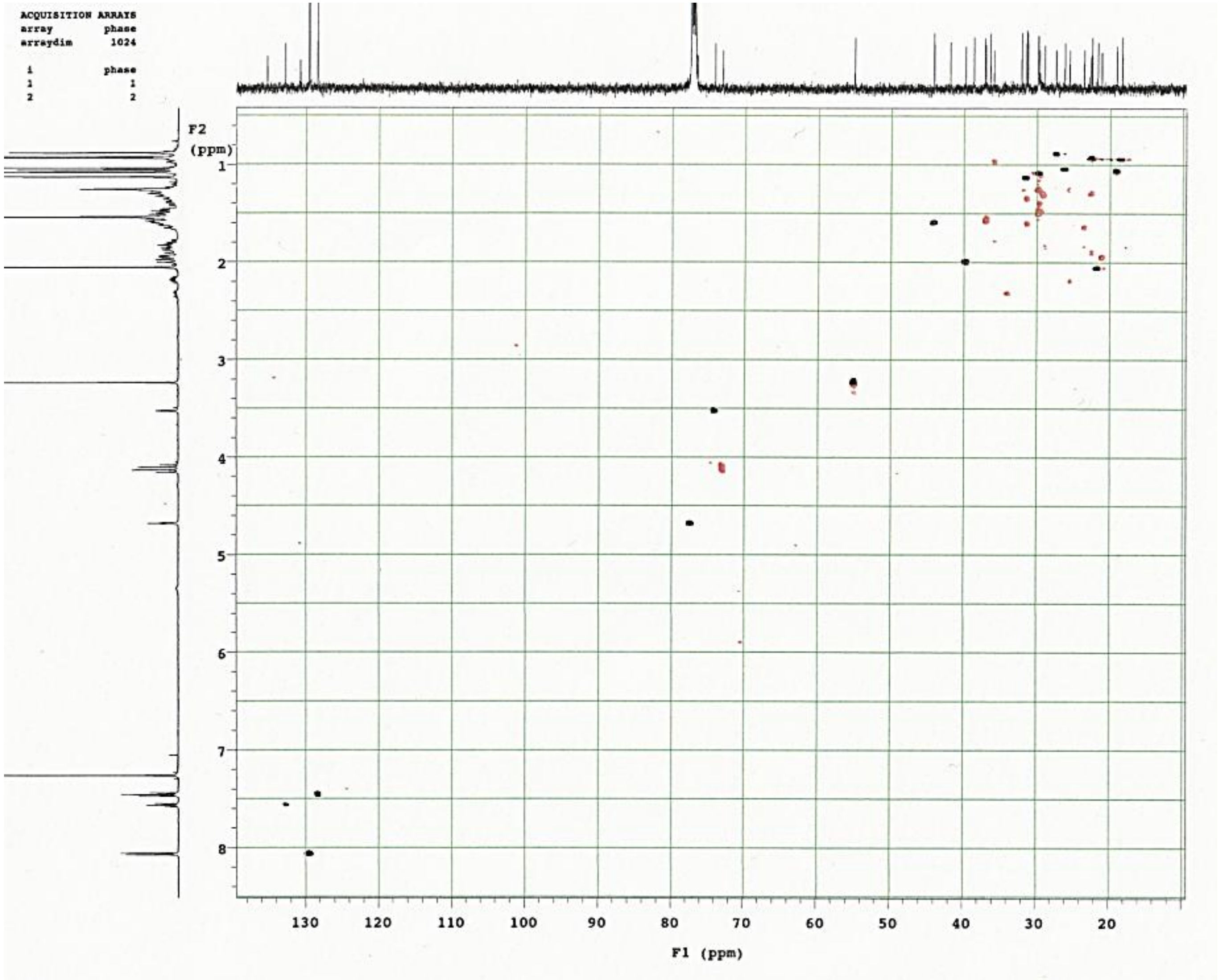

Figure S4. HMBC spectrum of compound **1**.

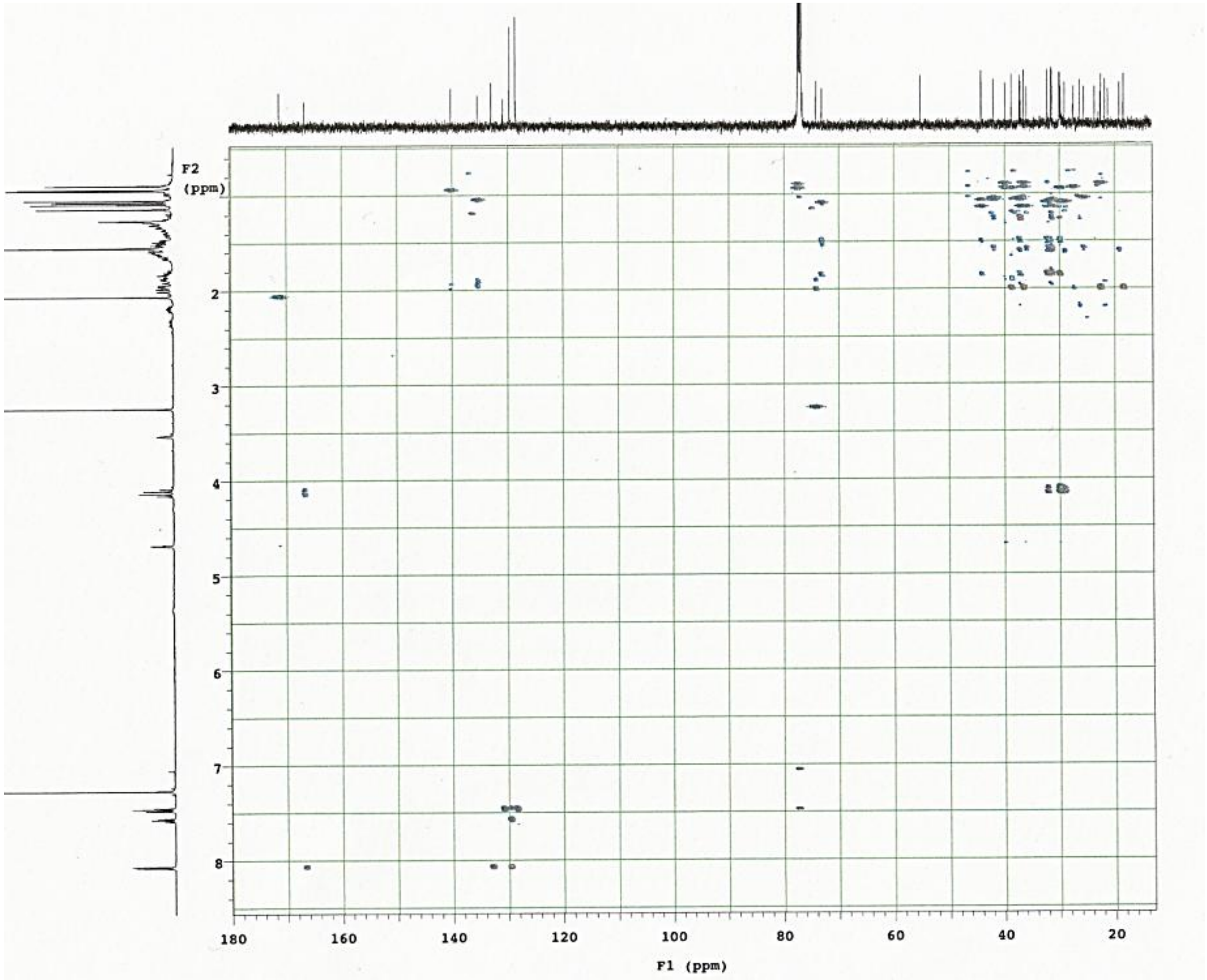

Figure S5. ^{1}H-^{1}H COSY spectrum of compound **1**.

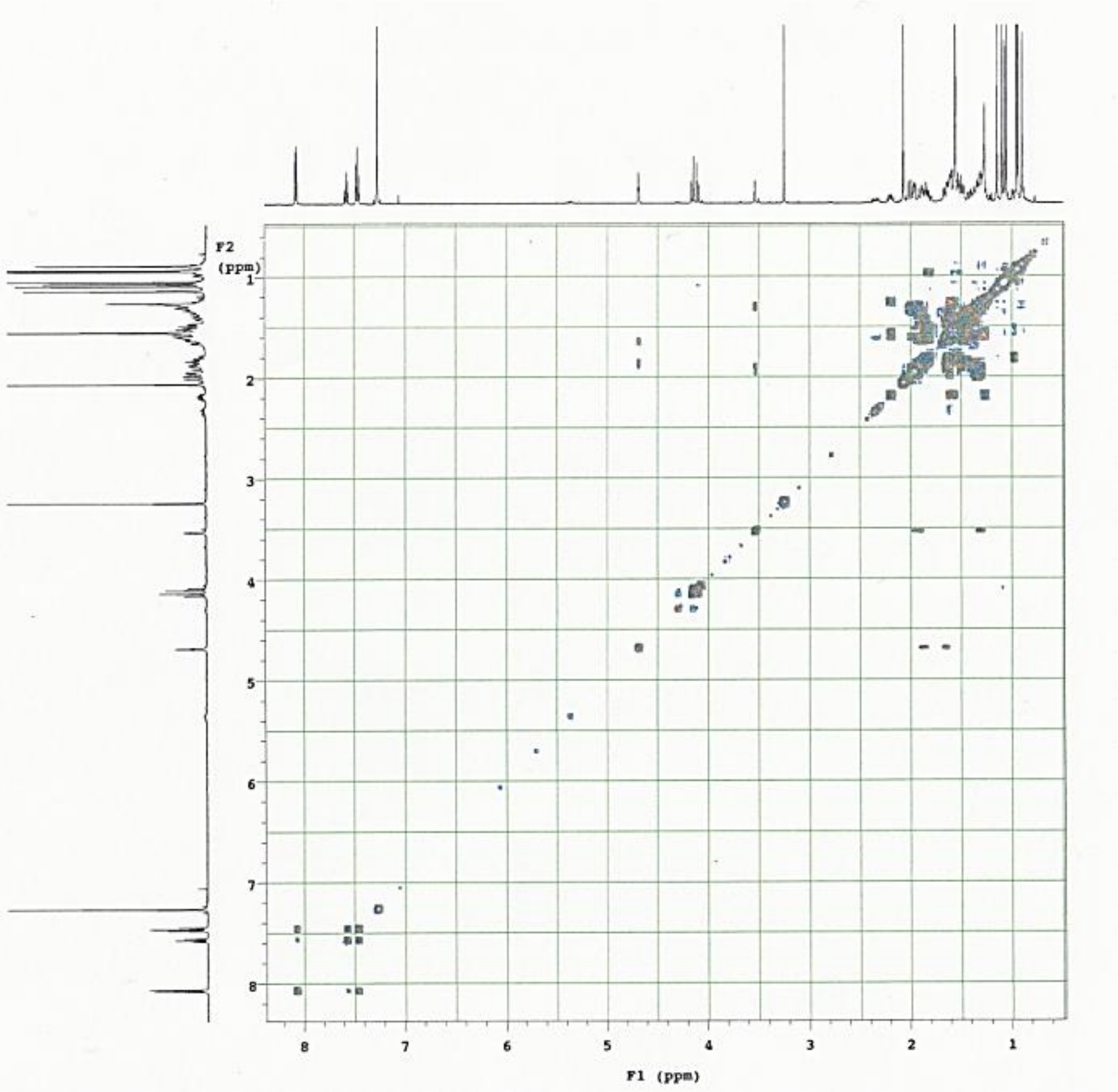

Figure S6. NOESYspectrum of compound **1**.

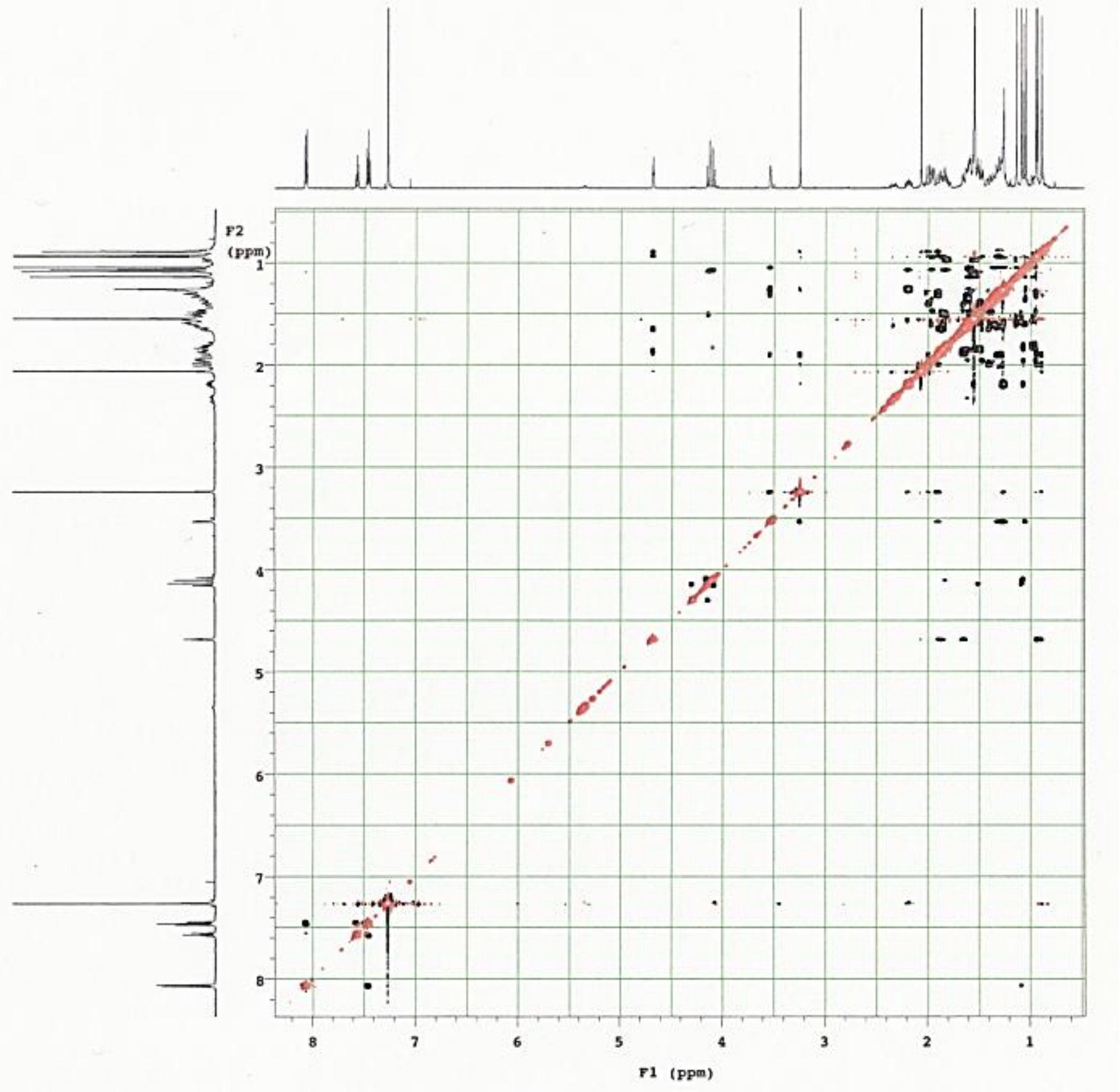

Figure S7. EIMS of compound **1**.

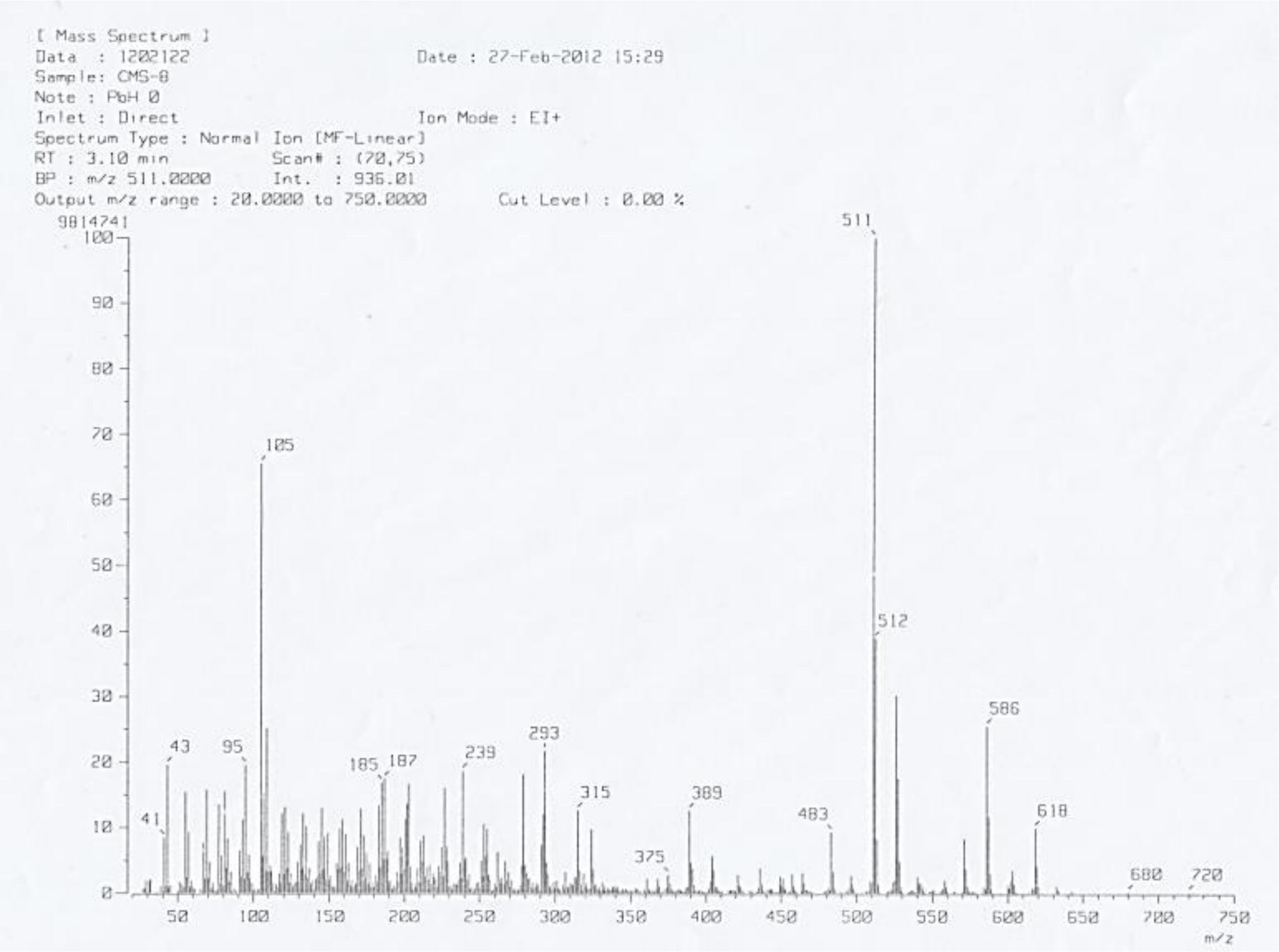

Figure S8. HREIMS of compound **1**.

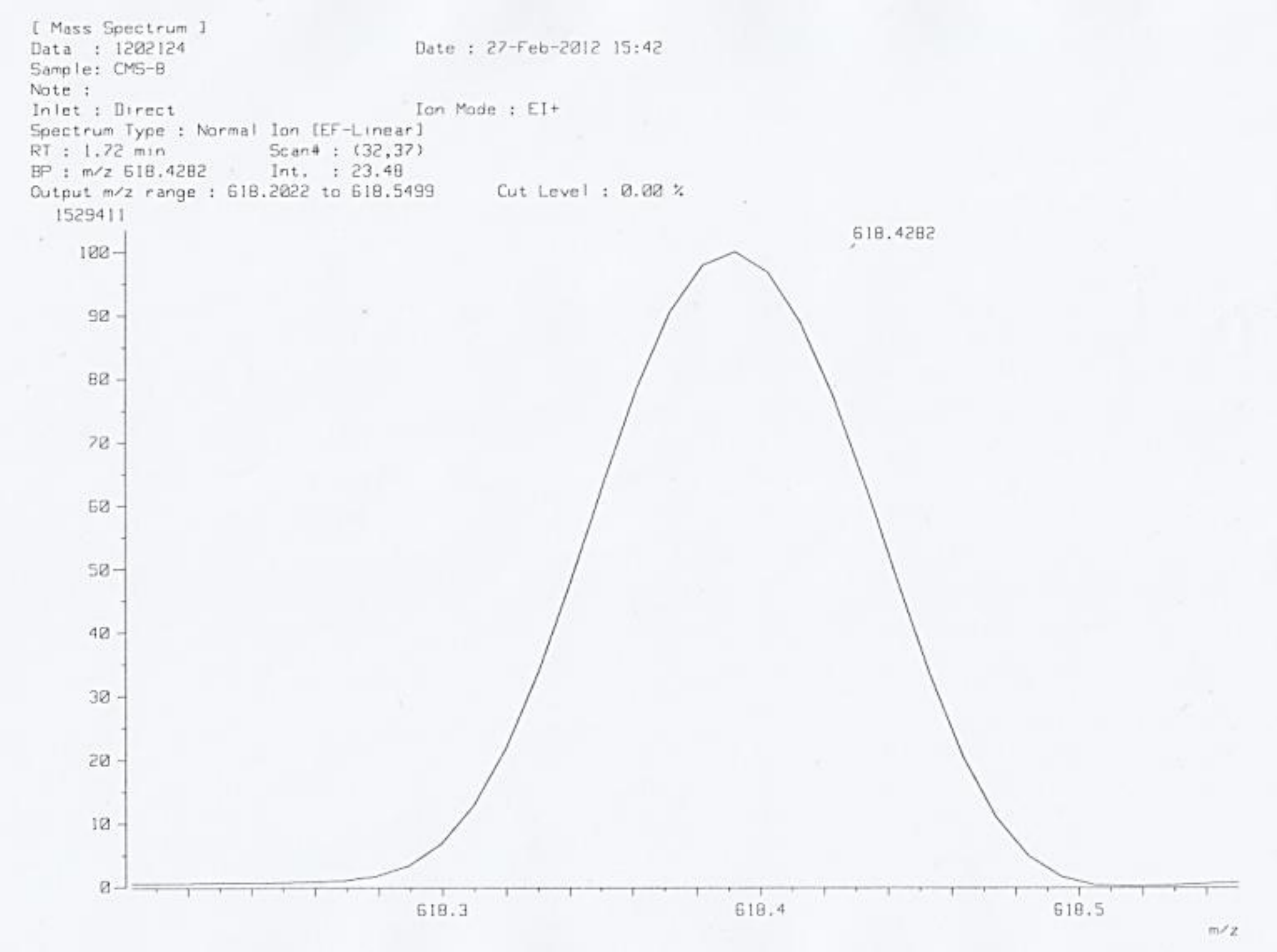

Table S1. ^{1}H (500 MHz) and ^{13}C (125 MHz), ^{1}H-^{1}H COSY, NOESY, and HMBC NMR spectroscopic data of compounds **1**.

**Position**		**δ_H (*J* in Hz)**	**^{1}H-^{1}H COSY**				**NOE**			**δ_C, type**	**HMBC (H to C)**					
1	α	1.39, m	1β	2α	2β		5α			29.7, CH_2	3	25				
	β	1.45, m	1α	2α	2β											
2	α	1.64, m	1α	1β	2β	3β				23.4, CH_2	1β					
	β	1.85, m	1α	1β	2α	3β	24	25								
3		4.68, t (2.8)	2α	2β			23	24		77.2, CH	23	24				
4										36.2, C	3	5α	23	24		
5		1.99, dd (12.5, 1.1)	6α	6β			1α	15α	23	39.7, CH	3	24	25			
6	α	1.89, m	5α	6β	7β					22.4, CH_2	5α					
	β	1.30, m	5α	6α	7β		24	25								
7		3.53, brs	6α	6β			15β	26		74.0, CH	5α	6β	7-O<u>Me</u>			
8										135.2, C	6β	11	26			
9										140.0, C	22	25				
10										38.5, C	2α	5α	6β	24	25	
11		1.95, m	12α	12β						21.0, CH_2						
12	α	1.34, m	11	12β						31.2, CH_2						
	β	1.60, m	11	12α			26									
13										37.0, C	15α	15β	18	19α	26	27
14										41.7, C	15α	16	26	27		
15	α	2.18, m	15α	16			5α	27		25.4, CH_2	16	26				
	β	1.25, m	15α	16			7β									
16		1.56, m	15α	15β						36.9, CH_2	28					
17										31.1, C	15β	19α	28			
18		1.59, m	19α	19β			26	28		44.0, CH	19α	27	28			
19	α	1.84, m	18β	19β						28.8, CH_2	18	29a	29b	30		
	β	1.30, m	18β	19α			28									
20										31.9, C	21	29a	29b	30		
21		1.51, m	22α	22β	27					29.5, CH_2	29a	29b	30			

Table S1. *Cont.*

Position		δ_H (*J* in Hz)	^{1}H-^{1}H COSY			NOE				δ_C, type	HMBC (H to C)		
22	α	1.79, m	21	22α	22β	27				35.7, CH_2	18	28	
	β	0.97, m	21	22α	22β								
23		0.88, s				3	5			27.2, CH_3	5	24	
24		0.93, s				2β	3	6β	25	22.3, CH_3	23		
25		0.94, s				2β	6β	26	24	18.2, CH_3	5		
26		1.04, s				4β	12β	18	25	26.1, CH_3	15α	15β	
27		1.06, s				15α	22α	29a	29b	18.9, CH_3	12β	18	
28		1.13, s				18	19β	30		31.2, CH_3			
29	a	4.15, d (10.8)				27				73.0, CH_3	19α	21	30
	b	4.11, d (10.8)				27							
30		1.08, s				28				29.7, CH_3	19α	29a	29b
3-O$\underline{\text{C}}$O										171.1, C	3	1′	
1′		2.06, s								21.5, CH_3			
29-O$\underline{\text{C}}$O										166.6, C	29a	29b	2″,6″
1″										130.7, C	3″, 5″		
2″, 6″		8.06, dd (7.4, 1.3)	3″, 5″	4″						129.5, CH	2″, 6″	3″, 5″	4″
3″, 5″		7.46, tt (7.4, 1.3)	2″, 6″	4″						128.3, CH	3″, 5″		
4″		7.57, tt (7.4, 1.3)	2″, 6″	3″, 5″						132.7, CH	2″, 6″		
7-OMe		3.24, s								55.0, CH_3			

Figure S9. ^{1}H-NMR spectrum of compound **2**.

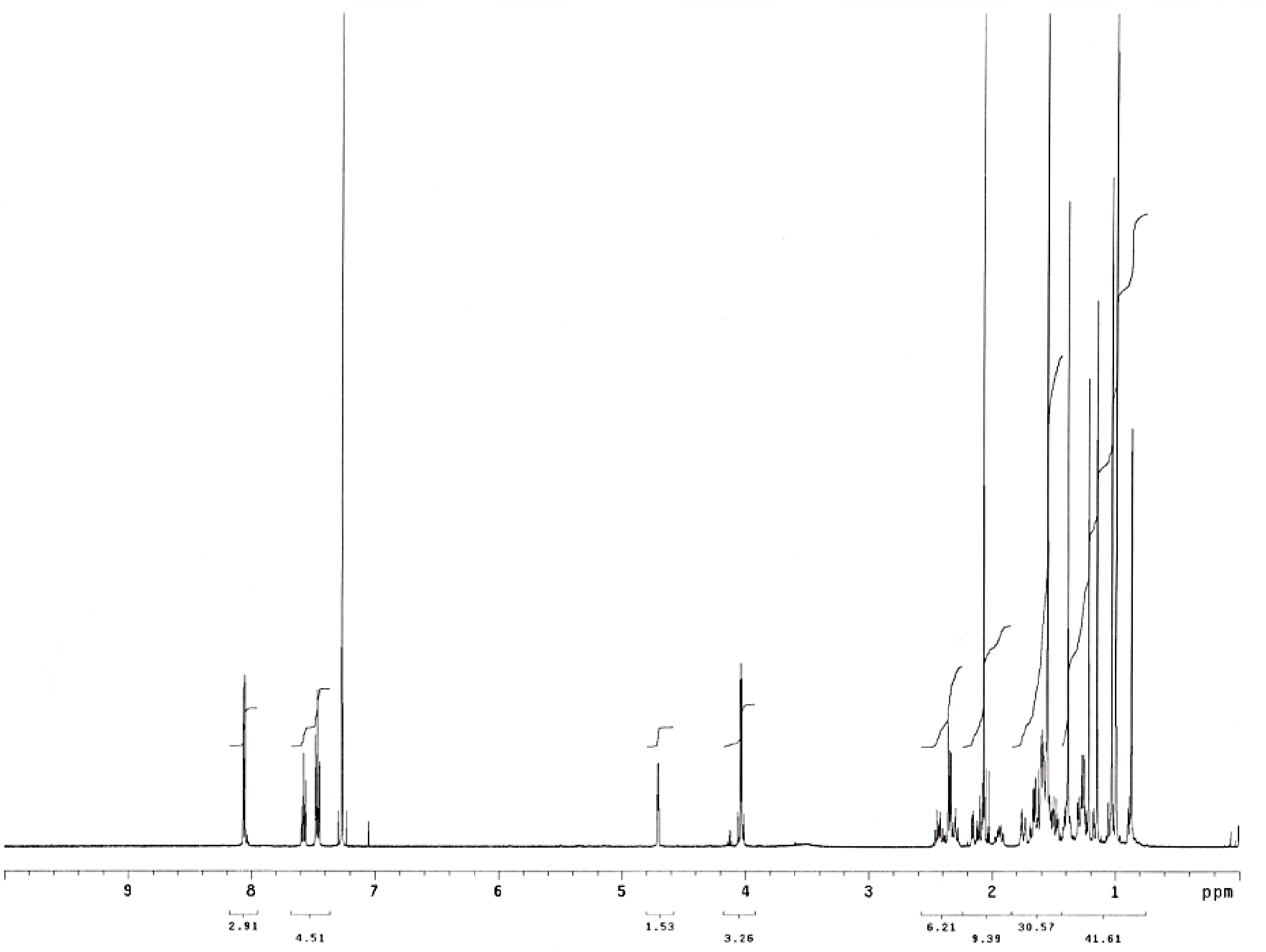

Figure S10. ^{13}C-NMR spectrum of compound **2**.

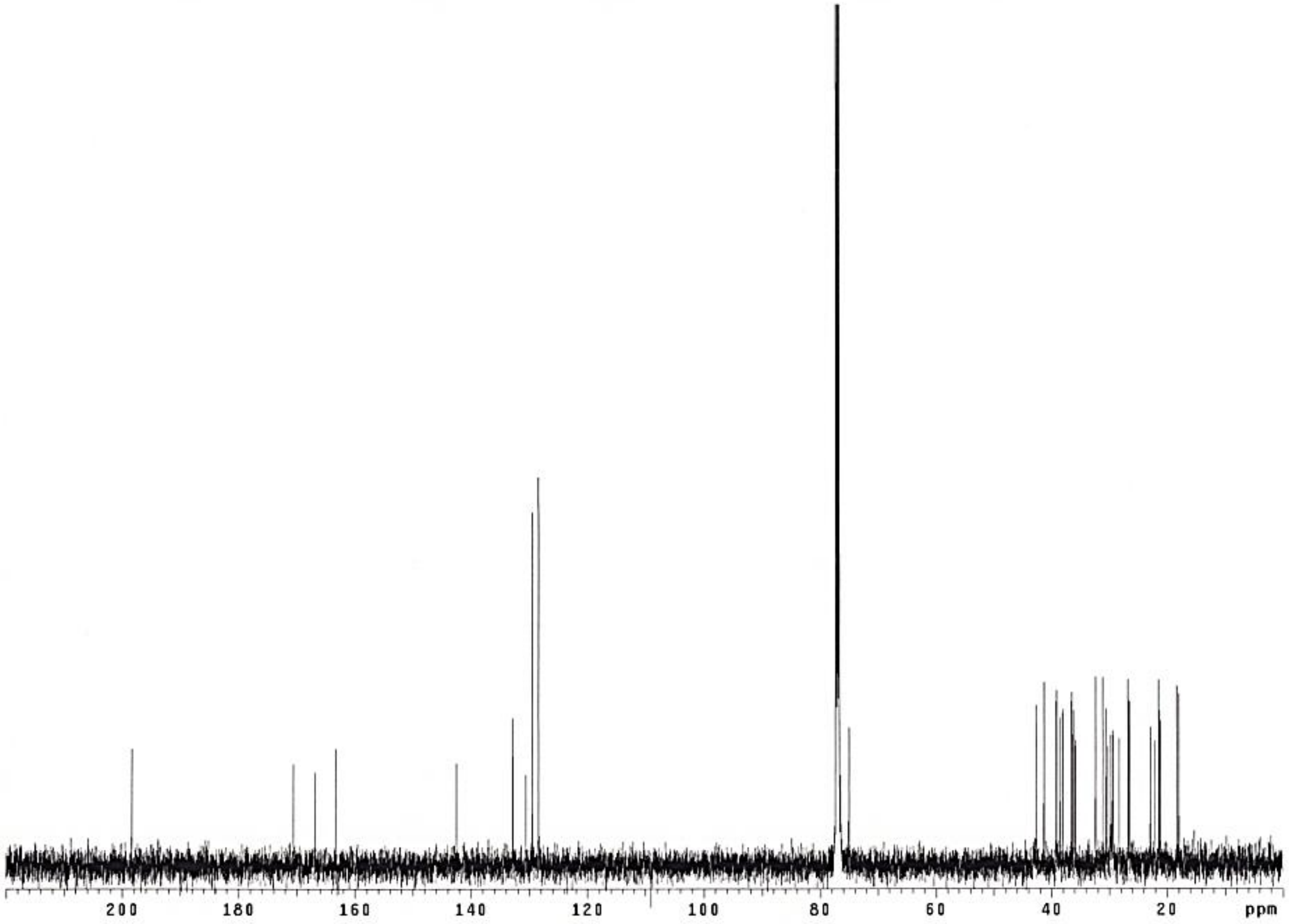

Figure S11. HSQC spectrum of compound **2**.

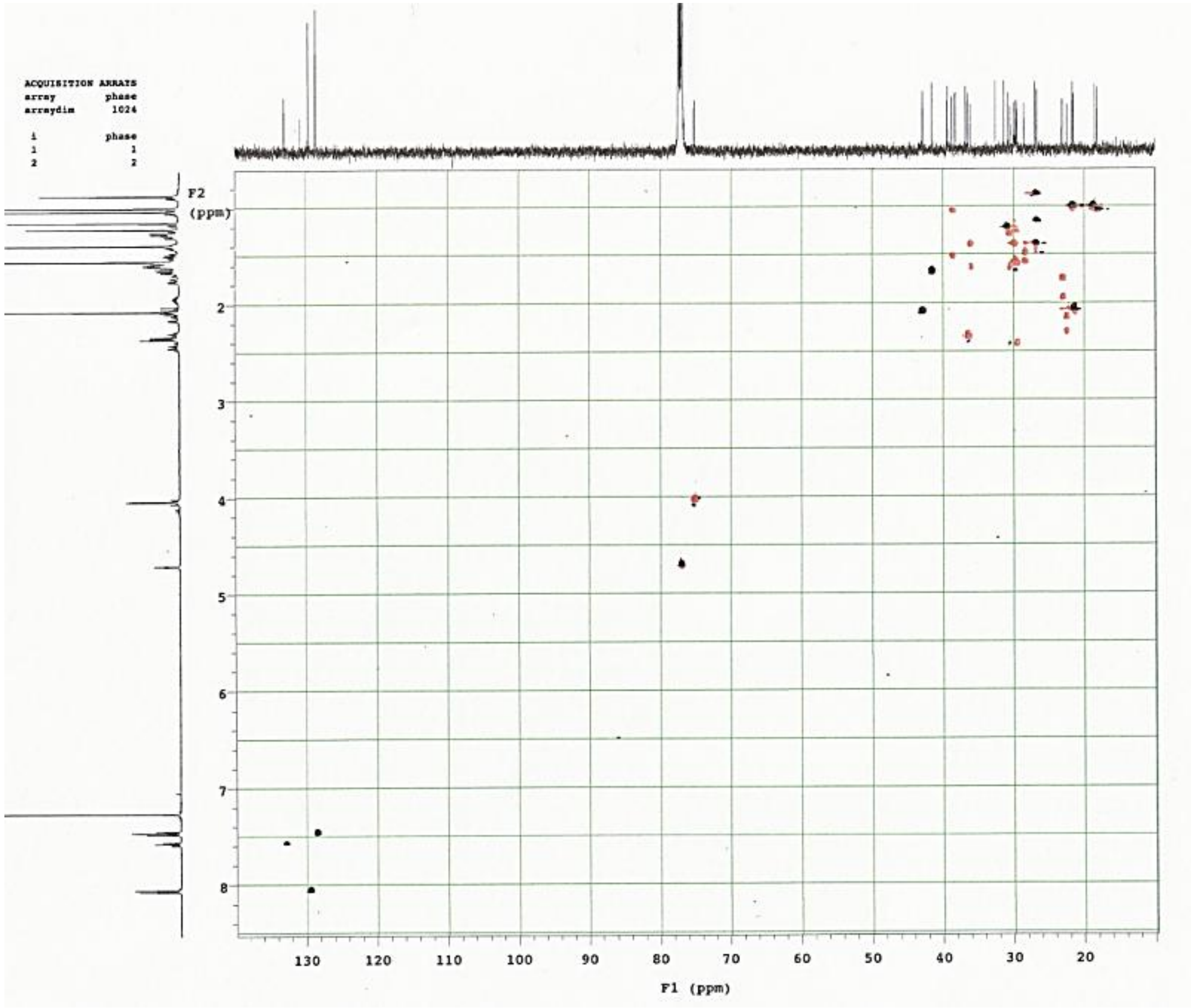

Figure S12. HMBC spectrum of compound **2**.

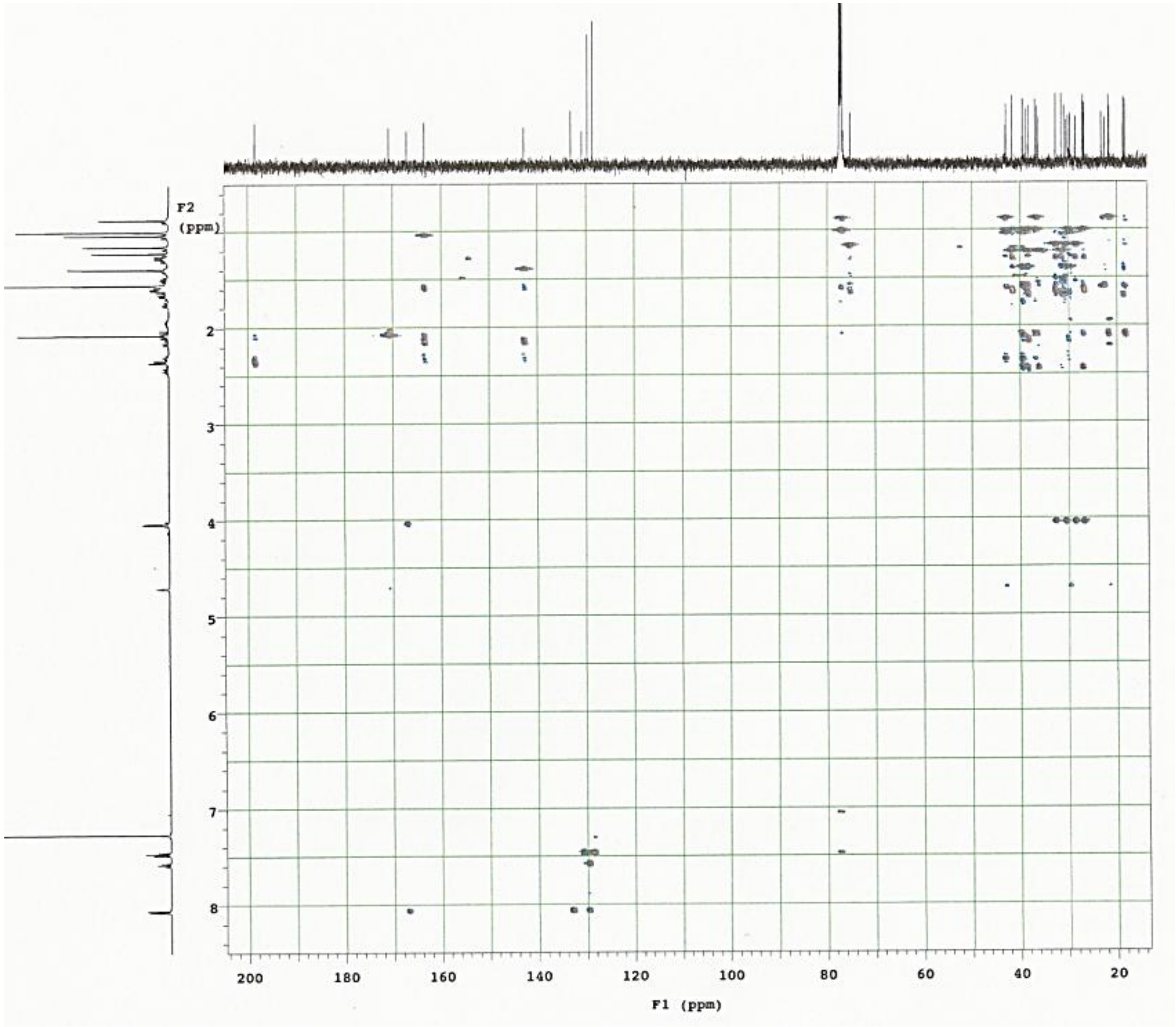

Figure S13. ^1H-^1H COSY spectrum of compound **2**.

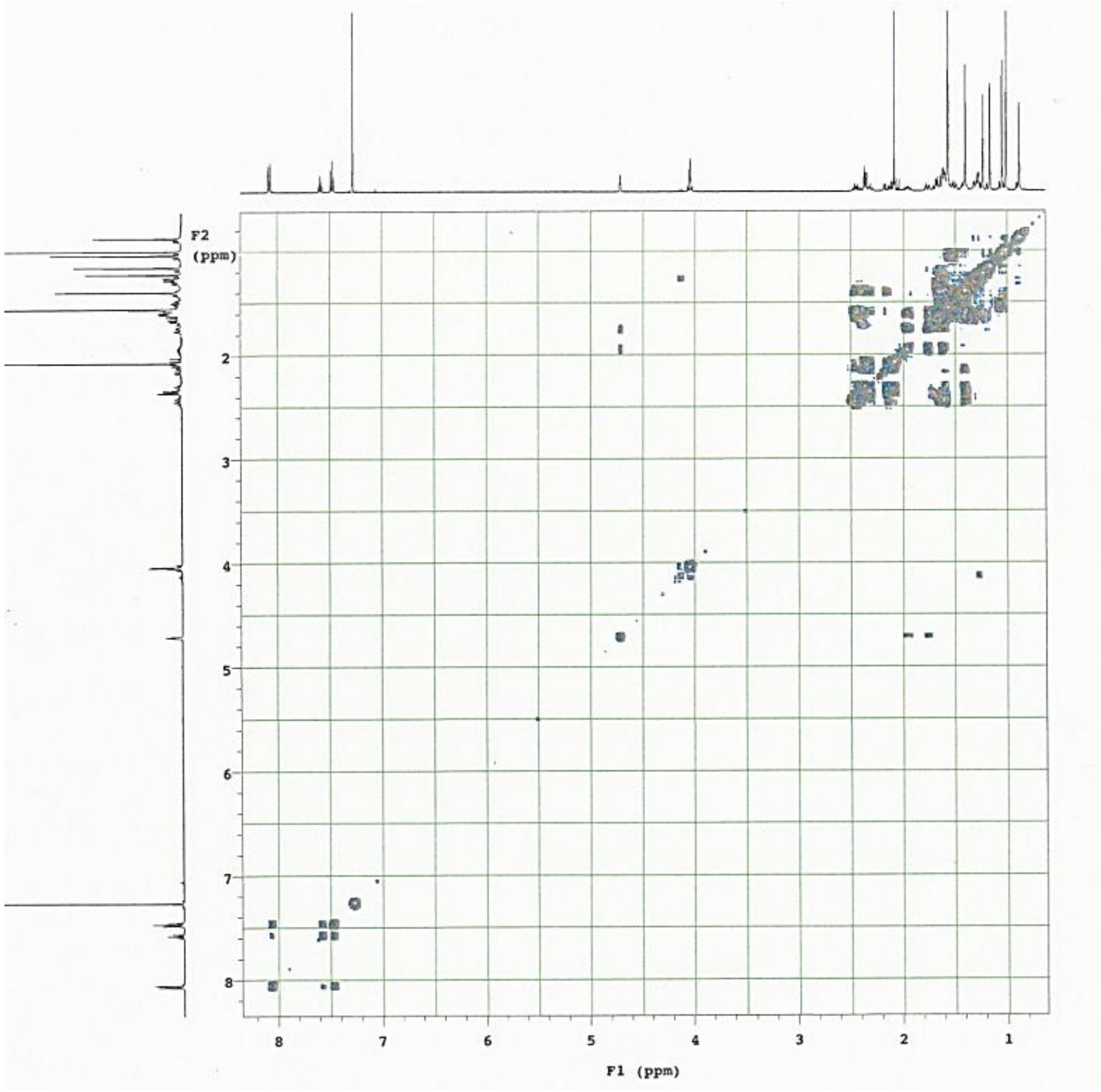

Figure S14. NOESY spectrum of compound **2**.

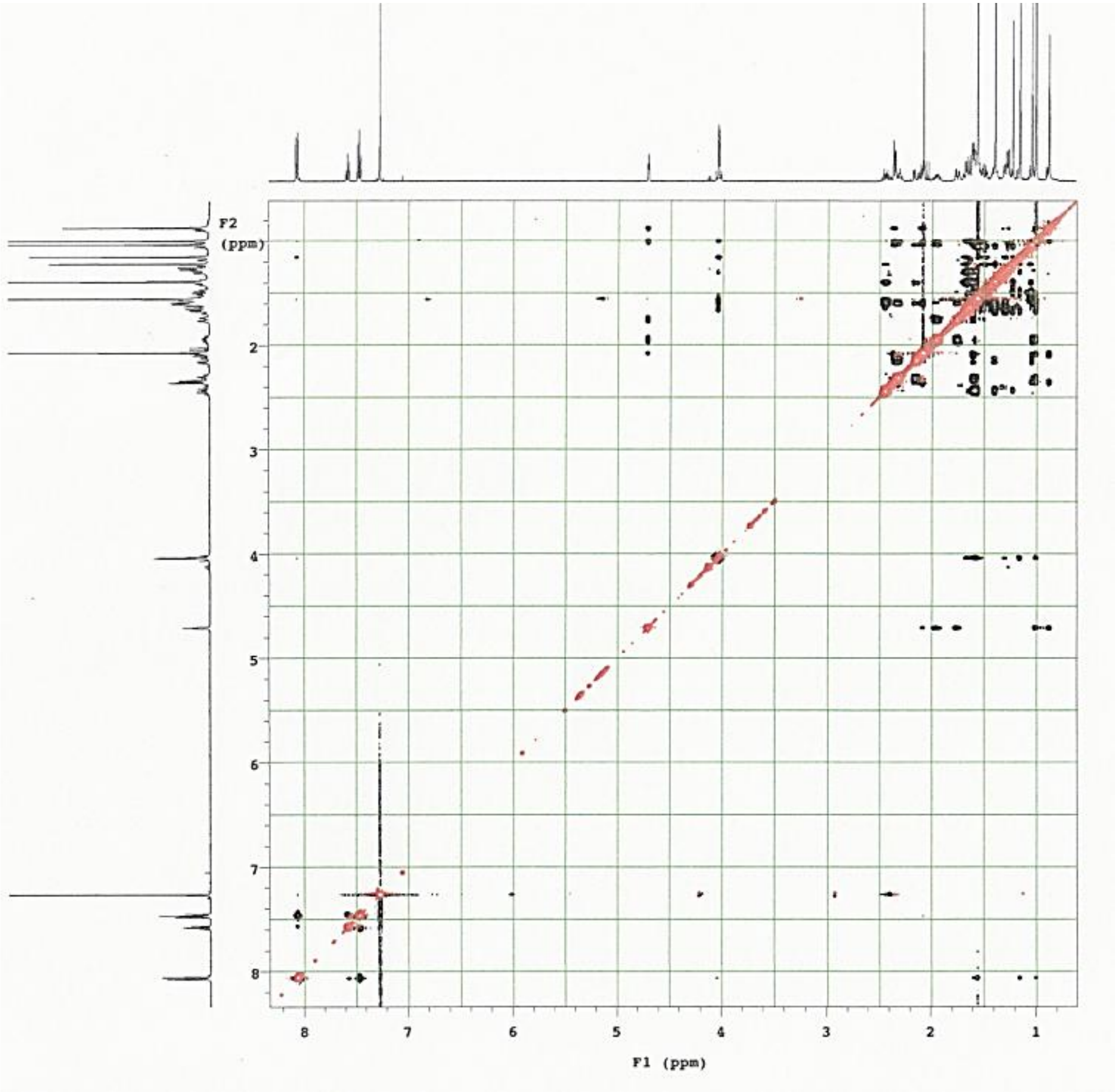

Figure S15. EIMS of compound **2**.

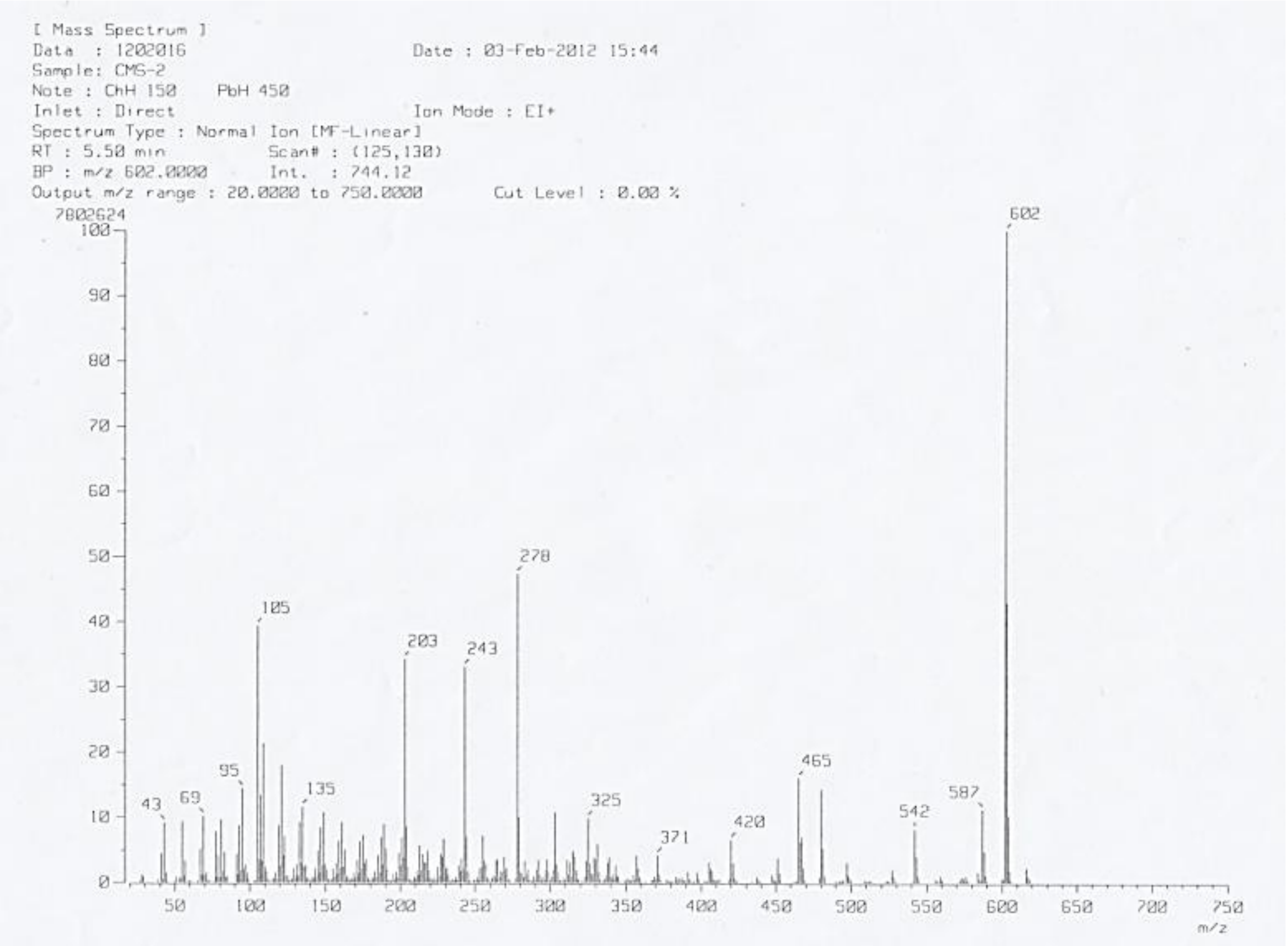

Figure S16. HREIMS of compound **2**.

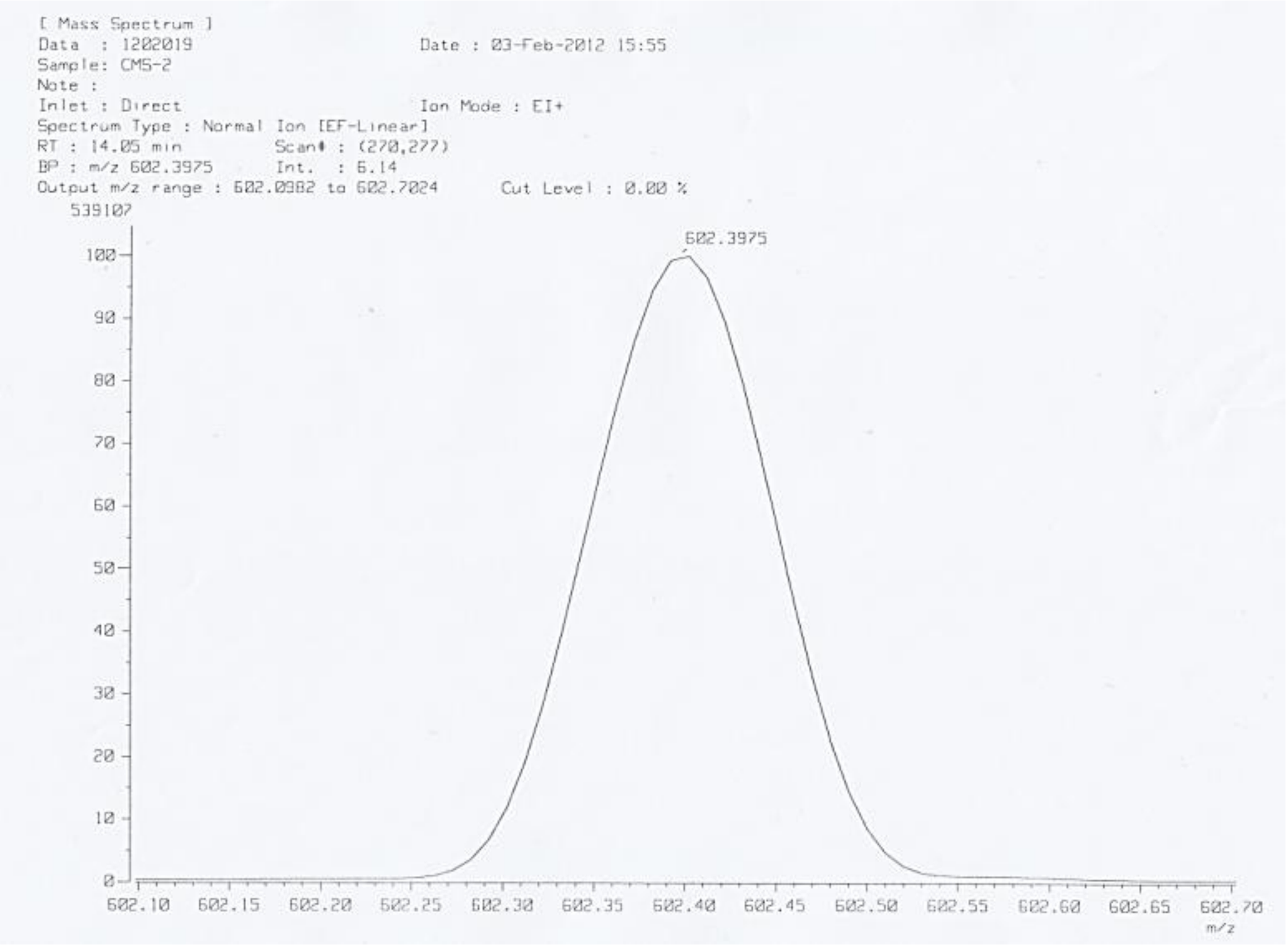

Table S2. ^{1}H (500 MHz) and ^{13}C (125 MHz), ^{1}H-^{1}H COSY, NOESY, and HMBC NMR spectroscopic data of compounds **2**.

**Position**		**δ_H (*J* in Hz)**	**^{1}H-^{1}H COSY**				**NOE**		**δ_C, type**	**HMBC (H to C)**						
1	α	1.97, m	1β	2α	2β		2″, 6″,		31.8, CH_2	3	25					
	β	1.58, m	1α	2α	2β		11									
2	α	1.87, m	1α	1β	2β	3β			23.1, CH_2	1α						
	β	1.98, m	1α	1β	2α	3β	24	25								
3		4.82, brd (3.2)	2α	2β			23	24	78.8, CH	1β	23	24				
4									37.6, C	3β	5α	23	24			
5		1.94, m	6α	6β			27		43.9, CH	1β	3β	6α	7	23	24	25
6	α	2.14, brt (5.0)	5α	6β	7		23		23.7, CH_2	5α						
	β	2.08, m	5α	6α	7		24	25								
7		5.06, brd (5.9)	6α	6β			15β		119.4, CH	5α	6α	6β				
8									142.3, C	6α	11	15α	15β	26		
9									145.8, C	7	25					
10									36.4, C	1β	2α	2β	5α	6α	11	25
11		5.29, brd (5.9)	12α	12β			1β		114.8, CH	12α						
12	α	2.08, m	11	12β			19α		39.1, CH_2	18β	27					
	β	1.79, m	11	12α			18β	26								
13									37.5, C	11	12α	18β	19α	26	27	
14									40.4, C	12α	15α	26	27			
15	α	1.63, m	15β	15α	16β		27		27.6, CH_2	16α	26					
	β	1.42, m	16α	16β	15α		7									
16	α	1.76, m	15α	15β	16β				37.2, CH_2	15α	28					
	β	1.49, m	15α	15β	16α											
17									31.9, C	28						
18		1.68, m	19α	19β			12β	26	45.1, CH	16α	19α	27	28			
19	α	1.76, m	18β	19β			12α		29.6, CH_2	18β	21	29a	29b	30		
	β	1.30, m	18β	19α												
20									29.9, C	29a	29b	30				
21		1.63, m	22α	22β					30.1, CH_2	19α	22α	29a	29b	30		

Table S2. *Cont.*

Position		δ_H (*J* in Hz)	^{1}H-^{1}H COSY		NOE				δ_C, type	HMBC (H to C)			
22	α	1.89, m	21	22β					33.0, CH_2	16α	18β	21	28
	β	0.95, m	21	22α	26								
23		0.90, s			3β	6α	25		28.0, CH_3	3β	5α	24	
24		1.03, s			2β	3β	6β	23	21.6, CH_3	3β	23		
25		1.01, s			2β	6β	24	26	21.1, CH_3	1β	5α		
26		0.94, s			12β	18β	22β	25	21.2, CH_3	15α			
27		1.03, s			5α	15α	29a	29b	19.9, CH_3	12α	12β	18β	
28		1.11, s							31.4, CH_3	16α	18β		
29	a	4.34, d (10.7)	29b		27				74.2, CH_2	19α	21	30	
	b	4.12, d (10.8)	29a		27								
30		1.16, s							31.3, CH_3	29a	29b		
3-O<u>C</u>O									171.1, C	3	1′		
1′									123.1, C	3′, 5′			
2′,6′		7.85, dd (8.4, 2.8)	3′, 5′		1α				131.9, CH	2′,6′			
3′, 5′		6.84, dd (8.4, 2.8)	2′,6′						115.3, CH	3′, 5′	4″-OH		
29-O<u>C</u>O									168.5, C	29a	29b	2″, 6″	
1″									130.2, C	2″, 6″			
2″, 6″		8.04, dd (7.4, 1.4)	3″, 5″	4″					129.6, CH	2″, 6″	3″, 5″		
3″, 5″		7.46, tt (7.4, 1.4)	4″	2″, 6″					128.8, CH	2″, 6″			
4″		7.56, tt (7.4, 1.4)	3″, 5″	2″, 6″					133.6, CH	2″, 6″			
4″-OMe		7.49, brs											

Figure S17. ^{1}H-NMR supectrum of compound **3**.

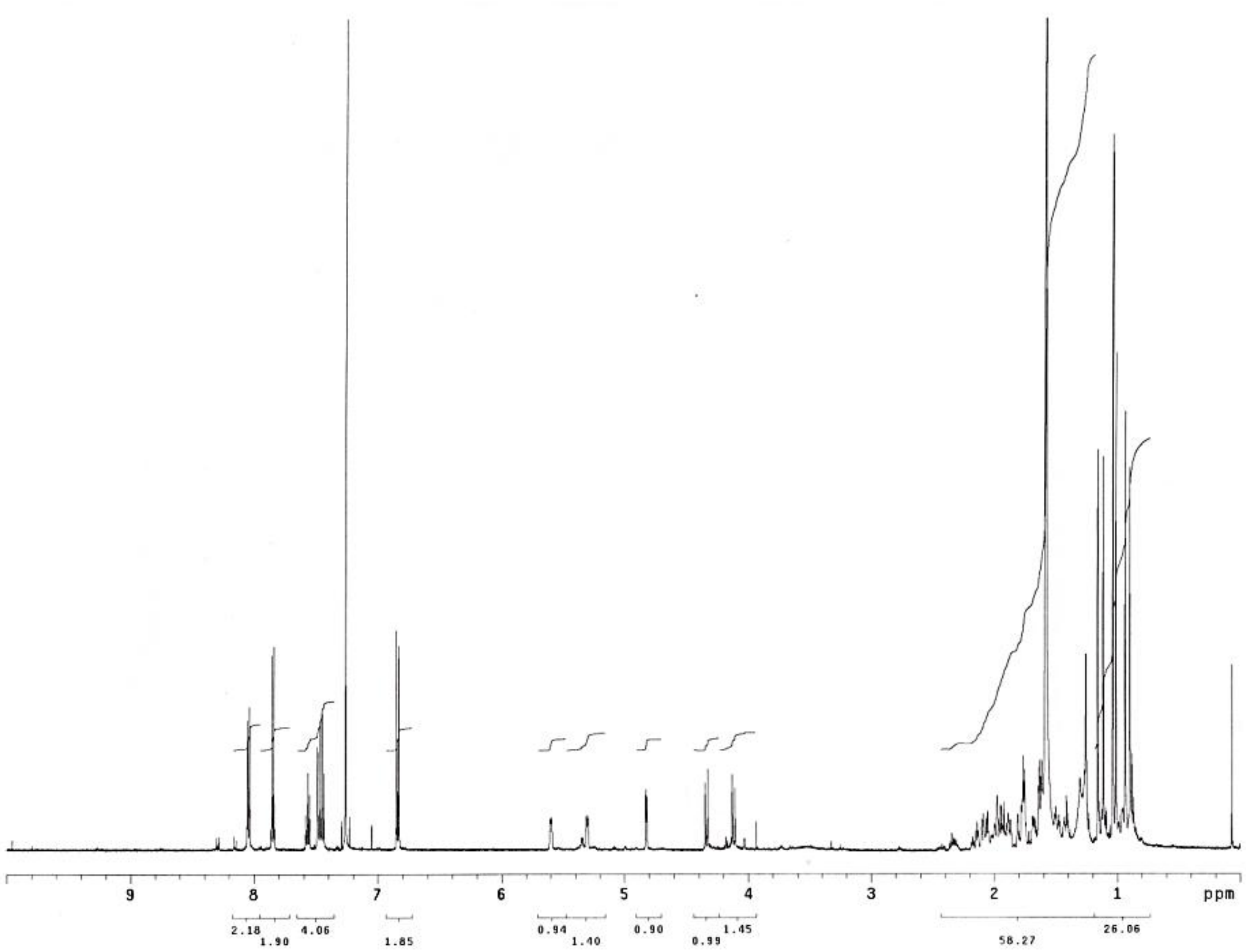

Figure S18. ^{13}C-NMR supectrum of compound **3**.

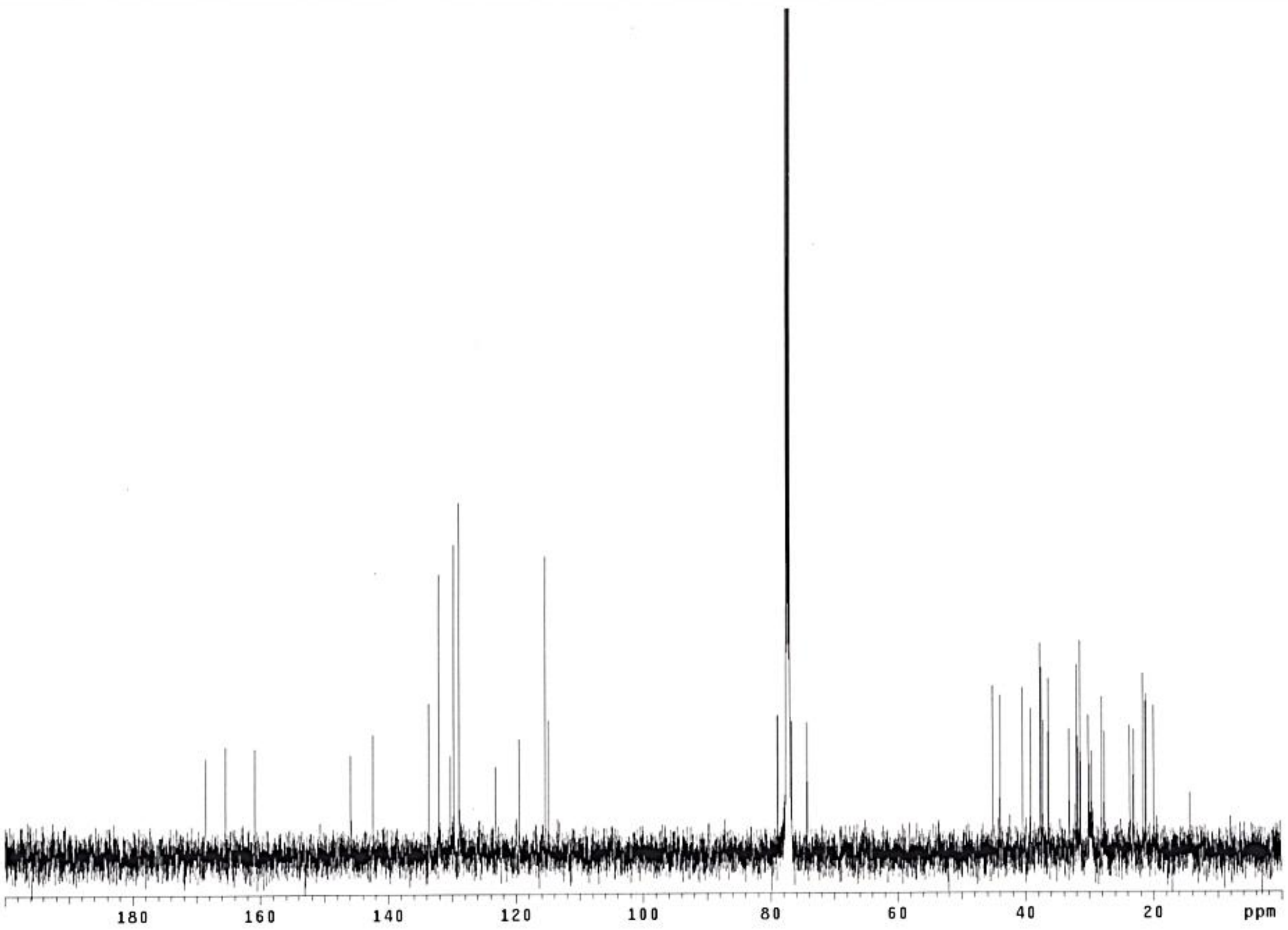

Figure S19. HSQC spectrum of compound **3**.

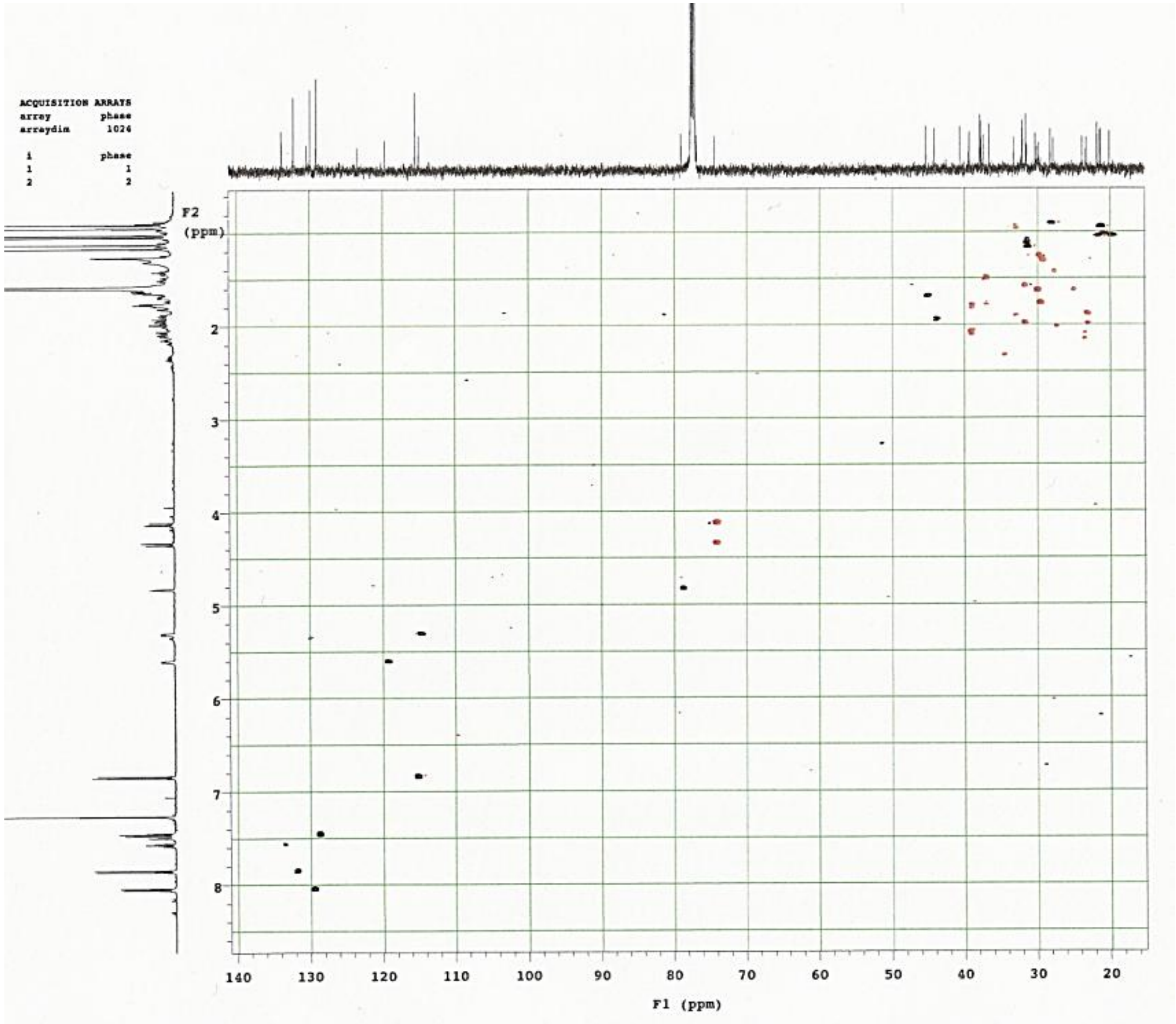

Figure S20. HMBC spectrum of compound **3**.

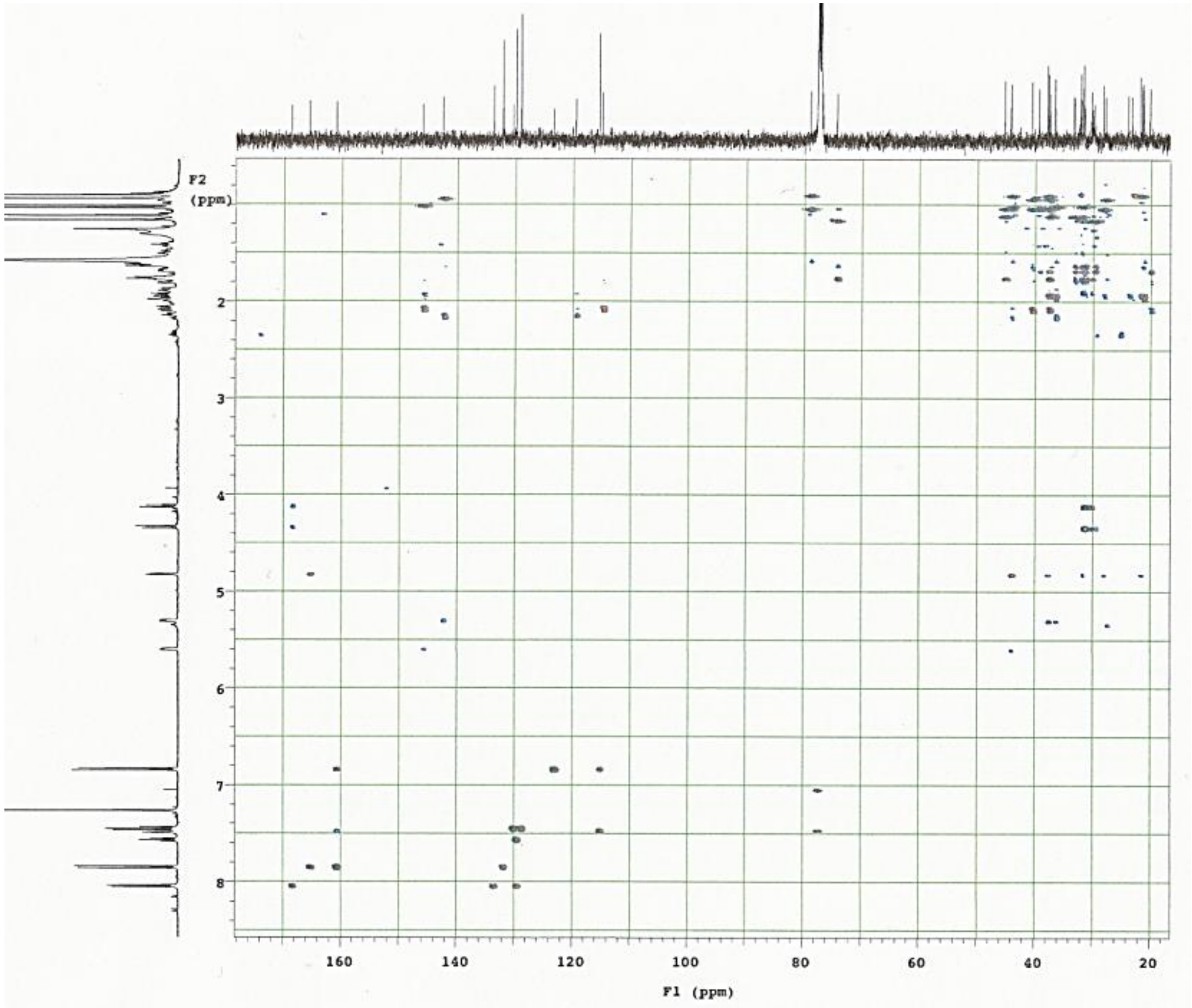

Figure S21. ^{1}H-^{1}H COSY spectrum of compound **3**.

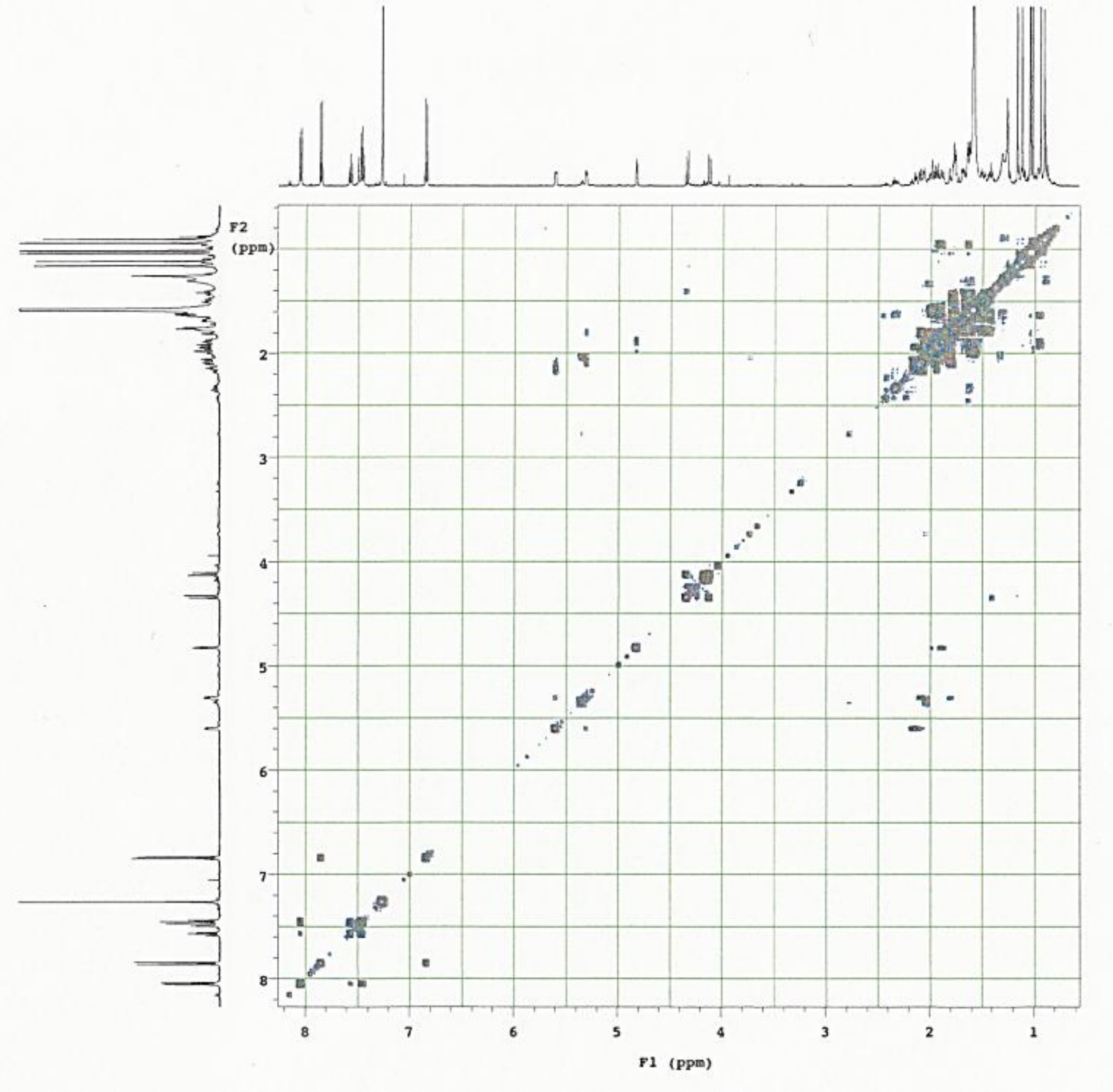

Figure S22. NOESY spectrum of compound **3**.

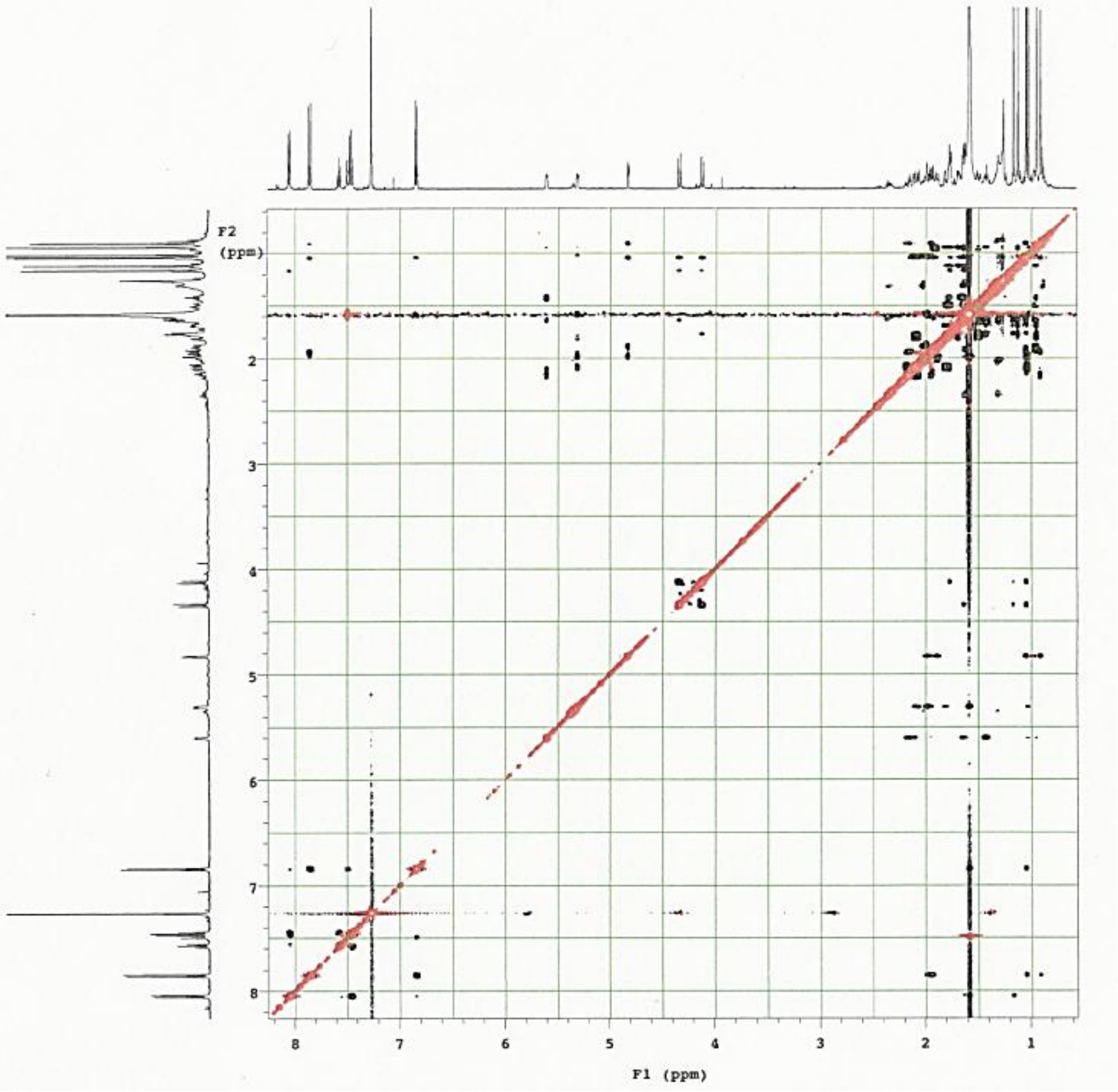

Figure S23. EIMS of compound **3**.

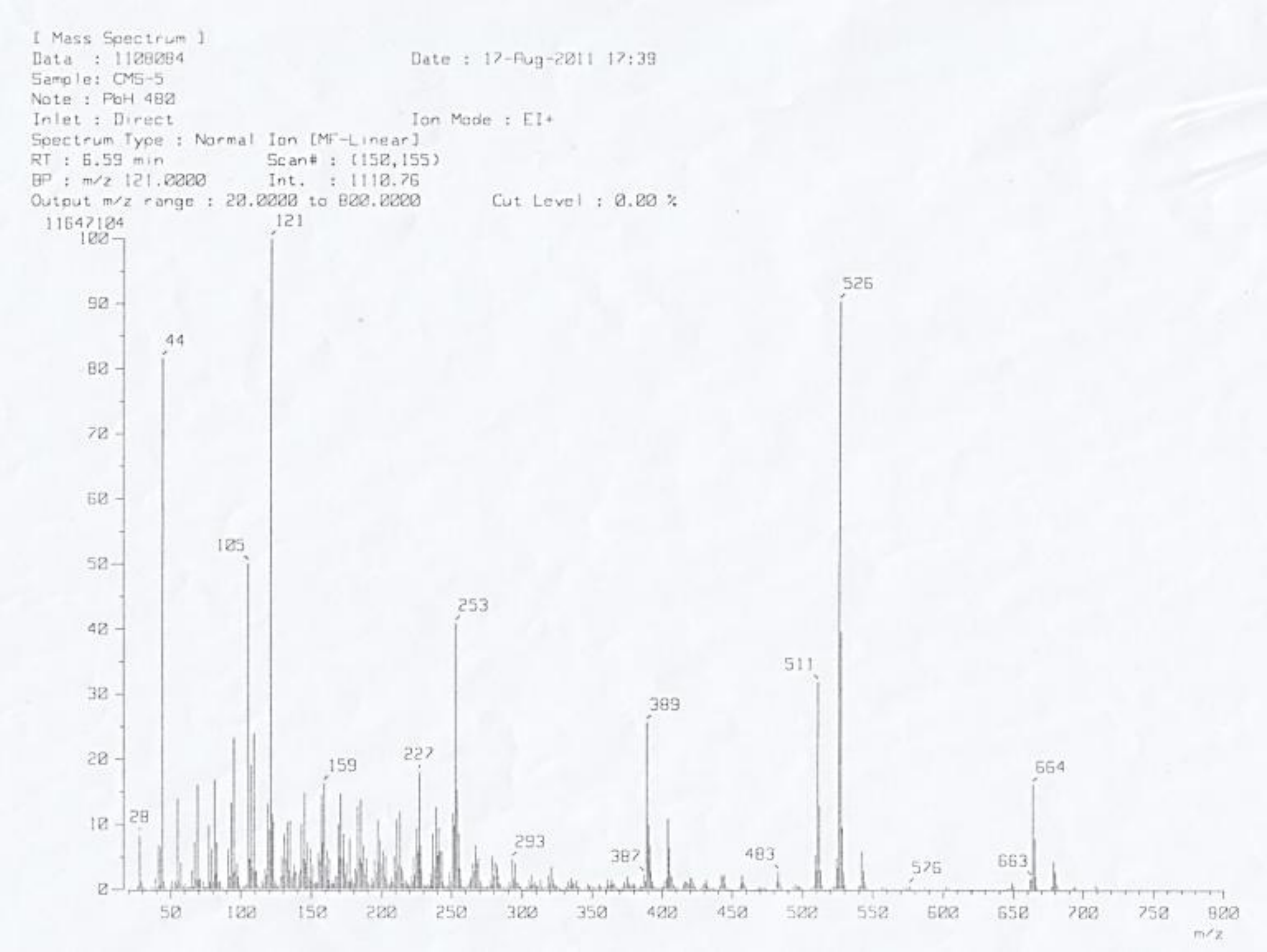

Figure S24. HREIMS of compound **3**.

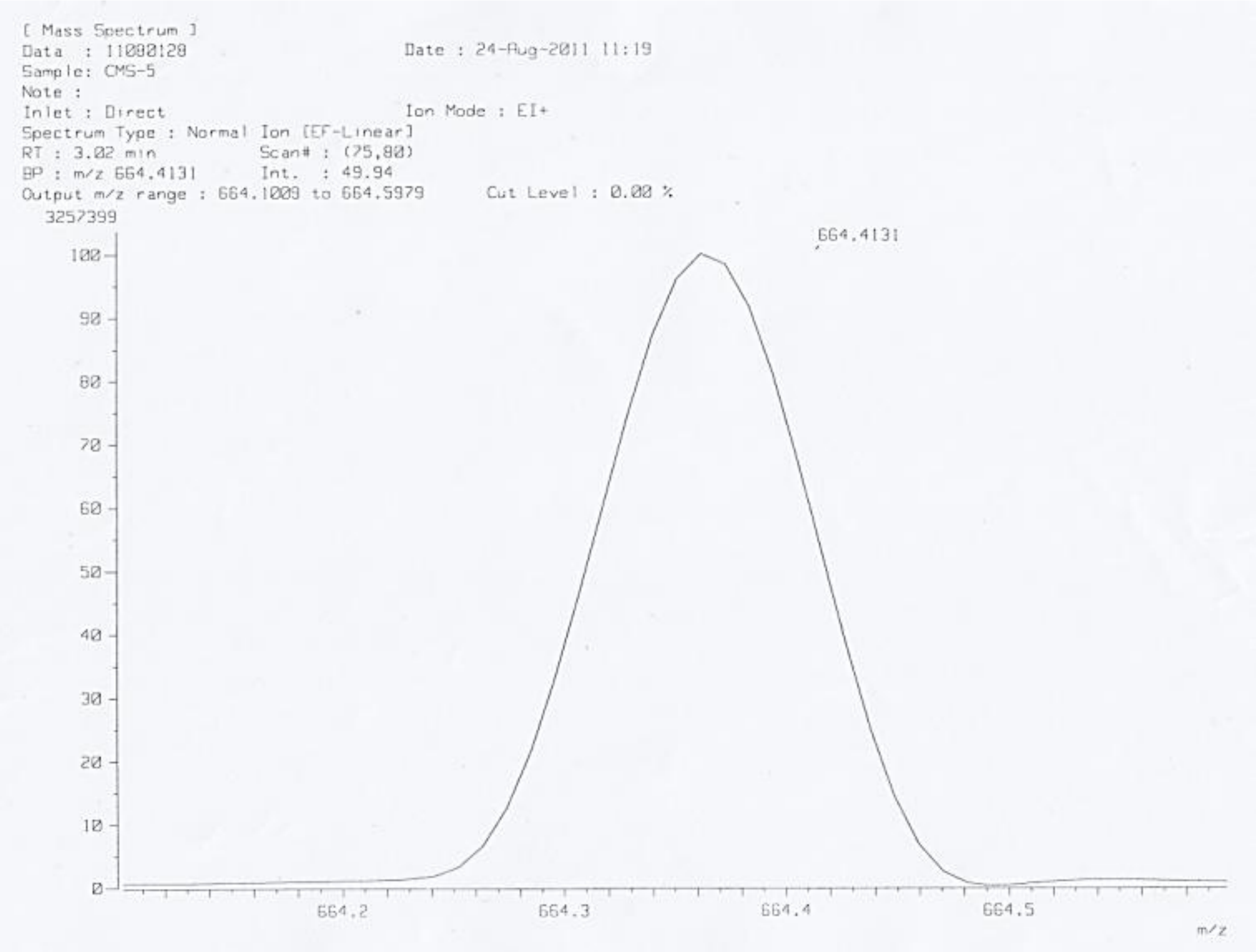

Supplement: Supplementary file 1 [file molecules-18-05568-s001.pdf]
